# Leisure activity engagement across adulthood predicts cognitive change after five years: Do gender and age matter?

**DOI:** 10.1017/S1355617722000510

**Published:** 2022-11-11

**Authors:** Sharon Sanz Simon, Seonjoo Lee, Yian Gu, Ashley Mensing, Diala Noofoory, Geneva M. Hidalgo Nazario, Reshma S. Babukutty, Yaakov Stern

**Affiliations:** 1 Cognitive Neuroscience Division, Department of Neurology, Columbia University, New York, NY, USA; 2 The Taub Institute for Research in Alzheimer’s Disease and the Aging Brain, Columbia University, New York, NY, USA; 3 The Gertrude H. Sergievsky Center, Columbia University, New York, NY, USA; 4 Department of Psychiatry and Biostatistics, Columbia University, New York, NY, USA; 5 The Department of Epidemiology, Joseph P. Mailman School of Public Health, Columbia University, New York, NY, USA

**Keywords:** aging, leisure activity, cognition, brain thickness, lifespan, cognitive reserve

## Abstract

**Objective:**

To examine the association between leisure activity (LA) frequency and cognitive trajectories over 5 years across adulthood, and whether gender and age moderate these associations.

**Method:**

A total of 234 cognitively healthy adults (21–80 years) completed a LA questionnaire at baseline and neuropsychological measures at baseline and after 5 years. Latent change score analysis was applied to generate latent variables estimating changes in different cognitive domains. For a secondary analysis, LA components’ scores were calculated, reflecting cognitive-intellectual, social, and physical activities. Regression analysis examined the association between baseline LA and cognitive change, and potential moderation of gender and age. In addition, we tested the influence of cortical gray matter thickness on the results.

**Results:**

We found that higher LA engagement was associated with slower cognitive decline for reasoning, speed, and memory, as well as better vocabulary across two time points. Regarding LA components, higher Social-LA and Intellectual-LA predicted slower rates of cognitive decline across different domains, while Physical-LA was not associated with cognitive change. Gender, but not age, moderated some of the associations observed. Our results remained the same after controlling for cortical gray matter thickness.

**Conclusions:**

We demonstrated a protective effect of LA engagement on cognitive trajectories over 5 years, independent from demographics and a measure of brain health. The effects were in part moderated by gender, but not age. Results should be replicated in larger and more diverse samples. Our findings support cognitive reserve hypothesis and have implications for future reserve-enhancing interventions.

## Introduction

Leisure activity (LA) can be defined as activity individuals engage in during free time (Sala et al., 2019), for enjoyment or well-being, independent from work or activities of daily living (Verghese et al., 2006). It has been suggested that engaging in LA may protect cognitive health in aging. A theoretical account for this association is the cognitive reserve hypothesis (Stern et al., 2018), which states that reserve can be built up through a combination of life experiences (Stern, 2012), such as education, occupation, and engaging in an active lifestyle consisting of physical activity, social relationships and cognitively demanding activities. These experiences may create a buffer against age-related cognitive decline and pathological processes by enhancing or compensating brain functioning and preserving cognitive ability.

Modifiable lifestyle factors have been suggested to protect brain health and influence dementia incidence, with an estimation that twelve factors account for 40% of dementia cases worldwide (Livingston et al., 2020). Among these factors are education/intellectual enrichment, physical activity, and social engagement; aspects that are commonly incorporated in LA and can be targeted in low-cost interventions (Belleville et al., 2019; Kivipelto et al., 2018; Leanos et al., 2020; Park et al., 2014). Despite the cumulative evidence of LA as a protective factor for dementia (Fratiglioni et al., 2020), in the context of healthy aging there is conflicting evidence on the association between LA engagement and cognitive decline. While cross-sectional studies show positive associations between cognitive function and LA engagement (Bielak et al., 2012; Casaletto et al., 2020; Gow et al., 2014; Mitchell et al., 2012), longitudinal data has not always supported this finding. Some studies found that LA at baseline was not associated with longitudinal change in cognitive performance in healthy older adults (Bielak et al., 2012; Gow et al., 2014; Mitchell et al., 2012). Others showed that LA engagement significantly slowed the rate of decline in different cognitive domains, such as language, executive functions (Ihle et al., 2019; Lifshitz-Vahav et al., 2017; Wang et al., 2013), processing speed (Ghisletta et al., 2006; Lovden et al., 2005; Small et al., 2012), and episodic memory (Lifshitz-Vahav et al., 2017; Richards et al., 2003; Small et al., 2012; Wang et al., 2013).

The inconsistency in the field may be attributed to the heterogeneity of the LA measures (Fratiglioni et al., 2020; Ghisletta et al., 2006). For instance, studies frequently select different LA items and focus on certain components independently (e.g., intellectual, physical, or social), while others evaluate them as an ensemble (Bielak et al., 2012; Gow et al., 2014). Although examining individual LA components is critical to better understanding its specific effects and potential mechanisms (Casaletto et al., 2020), doing so may not capture the diversity and potential interactions between the different LAs, which typically co-occur in real-life. LA participation can differ regarding duration, frequency, and intensity or level of effort, aspects that are not well captured since many studies investigate LA engagement based on binaiy responses (e.g., “yes vs. no” or “none vs. some”) (Armstrong et al., 2021; Gow, Mortensen, et al., 2012; Scarmeas et al., 2001).

It is worth mentioning that although physical activity and exercise are often used as interchangeable terms, they are not the same (Caspersen et al., 1985). Physical activity is defined as a broad term that refers to body movement produced by the contraction of skeletal muscles and increases energy expenditure. Exercise is a component of physical activity and refers to a planned, structured, and repetitive movement to improve or maintain physical fitness. In addition, physical LA has been measured based on self-report scales but also through objective metrics that track activity during the day/week. Our study focuses on a self-report measure of physical LA.

Another aspect that may account for inconsistencies across studies is the heterogeneity of cognitive outcomes adopted by the studies analyzing LA–cognition associations. Some studies have investigated this question utilizing a variety of cognitive domains such as executive functions, language, speed, memory, and global cognition (Wang et al., 2013; Yates et al., 2016). Other studies have focused on specific cognitive domains such as speed processing, attention (Ghisletta et al., 2006; Ihle et al., 2019; Lovden et al., 2005), or general cognitive ability based on a few tasks (Bielak et al., 2014; Gow et al., 2014; Gow et al., 2017). In addition, some studies have utilized only one test to assess a specific domain (Ihle et al., 2019; Lifshitz-Vahav et al., 2017), which does not reflect robust cognitive constructs and may depend on task specific characteristics (Bielak et al., 2012)

Moreover, a source of variability in the studies are the covariates included in the models. Socioeconomic status has not always been included as a covariate (Bielak et al., 2012; Casaletto et al., 2020), despite being a relevant factor that may influence the accessibility and time available to participate in LA. Reverse causality may be a critical aspect for conflicting evidence (Fratiglioni et al., 2020; Gow, Corley, et al., 2012; Gow, Mortensen, et al., 2012); for instance, premorbid intelligence and education may influence LA opportunities, choices, engagement, or maintenance/withdrawal from activities. It is also possible that when older adults perceive any cognitive change or difficulty this may affect LA participation.

To further understand the potential protective role of LA on cognitive functioning, it is optimal to account for brain measures, which would differentiate between cognitive reserve and brain maintenance mechanisms. According to the recent Reserve & Resilience framework definitions (https://reserveandresilience.com/definitions/), cognitive reserve is a brain property that allows for sustained cognitive performance in the face of age-related changes, brain insult, or disease. If LA functions as a cognitive reserve mechanism, LA would be associated with reduced cognitive decline given similar structural brain measurements. Considering the same framework, brain maintenance refers to the relative preservation of the brain over time as a determinant of preserved cognition in older age. If LA functions as a brain maintenance mechanism, LA would be associated with more preserved brain measures (Stern et al., 2018). While there are several brain measures sufficient to answer this theoretical question, cortical thickness is considered a good measure since it is less influenced by brain/head size, unlike others (e.g., brain volume). Therefore, to advance LA research, it is critical to analyze longitudinal data in a well characterized sample of cognitively healthy individuals, with robust measurement of LA and cognition, critical demographic covariates, and brain health measures.

Another research path, and potential source of inconsistencies across studies, is the moderation role of gender and age in activity-cognition associations. It is possible that the LA–cognition associations are influenced by demographics such as age and gender, which are known to influence cognitive functioning and can influence the way people are exposed to LA. A better understanding of moderators may be more precisely informative of when it is more critical to engage in LA, or if there is any age or gender specificity, an aspect that may inform recommendations and interventions.

Gender differences have not gained much attention in LA literature (Bielak, 2010; Hassing, 2020), although patterns of LA may differ between men and women given cultural differences in roles and occupational/career opportunities. Evidence shows gender differences in LA engagement and its association with cognitive functioning. In women, higher involvement in intellectual-cultural activities was associated with better cognitive ability, and higher engagement in domestic activities predicted steeper cognitive decline. In men, higher involvement in self-improvement activities (e.g., participation in clubs, studies and sports) was associated with better cognitive ability across different domains (Hassing, 2020). In addition, there is evidence that cognitively and socially engaging LA in midlife among men (Carlson et al., 2008), and intellectual-cultural activities among women (Crowe et al., 2003) reduces the risk of dementia 20–40 years later.

It remains unclear whether the association between LA engagement and cognitive decline varies as a function of age. It is hypothesized that engaging in cognitively stimulating activities might have the greatest impact at older ages (Bielak et al., 2012; Bielak, 2010; Bielak et al., 2014), when cognitive decline becomes more marked, and the general effects of lifestyle have had the greatest opportunity to accumulate. This hypothesis has found empirical support, but there is limited evidence across the range of adulthood. For instance, in a comparison of cohorts in their early twenties, forties, and sixties, a positive association between LAs and cognitive performance was observed only among the older population (Parslow et al., 2006). Similarly, a greater effect of physical activity was observed among older versus younger adults (Hillman et al., 2006; Ogino et al., 2019). Nevertheless, other groups did not find a greater effect of cognitive activity on cognition in older adulthood (Newson & Kemps, 2006; Salthouse et al., 2002).

Taking into account the aforementioned literature and methodological considerations, the aims of the current study were to: (1) examine the protective role of LA engagement on cognitive change, considering a 5-year span in cognitively healthy adults aged 21 through 80; (2) explore if specific components of LA (i.e., cognitive-intellectual, social, or physical) are the driver of the relationship between LA engagement and cognitive change; (3) observe whether the association between baseline LA and cognitive change is independent of cortical thickness; and (4) assess the moderating role of gender and age in the LA–cognition associations. Our main hypothesis is that LA is associated with slower cognitive decline across two time points regardless of demographics, and we expect this effect to be independent from brain thickness, supporting the cognitive reserve framework. In our exploratory analysis, we also expect to observe that LA components will predict cognitive change across two time points, but we do not anticipate a specific cognitive benefit to be associated with one activity modality. Regarding the role of moderators, we hypothesized that gender is a moderator of LA–cognition associations in the sense that LA engagement benefits cognitive functioning differently in men and women, however we do not anticipate specific cognitive benefit by gender when considering past research. In relation to age, we hypothesized that LA would be strongly associated with cognitive change at older ages, when cognitive decline becomes more pronounced, and effects of lifestyle have had the greatest opportunity to accumulate.

Our approach adds to existing knowledge by implementing methodological strengths, such as: an aggregate LA frequency score comprising of a range of common activities that reflect real-life experience; concomitantly addressing specific LA components defined based on previous works (Salthouse, 2006; Scarmeas et al., 2001; Verghese et al., 2003) and tested in a confirmatory analysis; robust measurement of cognitive abilities well established to change with aging (Habeck et al., 2016; Salthouse et al., 2015); inclusion of multiple cognitive tasks for each cognitive ability predefined statistically; use of latent change score modeling to calculate cognitive scores at baseline and follow-up; and controlling for critical demographics in addition to age and gender, such as education and SES. Critically, we are the first to test cognitive reserve vs. brain maintenance mechanisms when examining LA–cognitive change associations, and the exploration of gender and age moderation adds to the limited research on the potential moderators of these associations.

## Methods

### Sample characteristics

Participants derived from two ongoing studies at Columbia University Irving Medical Center: the Cognitive Reserve (CR) and the Reference Ability Neural Network (RANN) studies. The CR was designed to elucidate the neural underpinnings of cognitive and brain reserve (Stern et al., 2018), and RANN was designed to identify networks of brain activity uniquely associated with performance across the lifespan for four reference abilities: memory, reasoning, speed, and vocabulary (Habeck et al., 2016). This is a longitudinal study utilizing data from these two cohorts.

Both studies share similar recruitment and data collection procedures. Participants who met initial inclusion criteria (i.e., right-handed, native English speakers, no psychiatric/neurological disorders, and normal or corrected-to-normal vision) were evaluated with structured medical screening and neuropsychological evaluations to ensure there were no cognitive impairments, and neuropsychiatric conditions. In addition, information on lifestyle, such as engagement in LA, were collected. For inclusion, a score equal or greater than 130 was required on the Mattis Dementia Rating Scale (Mattis, 1988), and preserved functionality in the Blessed Activities of Daily Living scale (Blessed et al., 1968).

Follow-up evaluation occurred at a 5-year interval, when MRI scanning, cognitive, and functional measures were repeated. Performance on the cognitive battery at baseline or follow-up that was indicative of mild cognitive impairment (MCI) or dementia was grounds for exclusion. Diagnoses of MCI or dementia was determined in consensus between a neurologist and neuropsychologist who reviewed the medical records to adjudicate a diagnosis based on standard research criteria. The current analysis included 234 participants (age range 21–80 years), who were assessed at both baseline and follow-up, had cognitive data in both assessments, and completed leisure activity questionnaires at baseline. The studies were approved by the Institutional Review Board of the College of Physicians and Surgeons of Columbia University and was completed in accordance with Helsinki Declaration.

### Cognitive tasks

At baseline and follow-up, participants underwent a comprehensive neuropsychological (out-of-scanner) evaluation and performed several computerized cognitive tasks during the MRI exam (Gazes et al., 2021; Habeck et al., 2016; Salthouse et al., 2015). As per a previous study from our group (Salthouse et al., 2015), 12 measures were selected based on a factor analysis reflecting four domains: reasoning, processing speed, memory, and vocabulary. Previous works indicate that reasoning, speed, and memory decline with cognitive aging, while vocabulary tends to increase or remain stable over time (Gazes et al., 2021; Habeck et al., 2016; Salthouse, 2004; Salthouse, 2009, 2019; Stern et al., 2014). Each cognitive domain included three measures: *reasoning:* Wechsler Adult Intelligence Scale (WAIS-III) Matrix Reasoning, Letter-number Sequencing, and Block Design test (Wechsler, 1997); *speed*: WAIS-III Digit-symbol (digit coding test), Stroop Color Naming Test (Golden, 1975), and Trail Making Test (TMT)-A (time) (Reitan, 1978); *memory*: Selective Reminding Test (SRT); last trial, continuous long-term retrieval and last retrieval (Buschke & Fuld, 1974); *vocabulary*: WAIS-III Vocabulary test, the Wechsler Test of Adult Reading (WTAR) (Wechsler, 2001), and the American National Adult Reading Test (AMNART) (Grober et al., 1991).

As described previously (Gazes et al., 2021; Habeck et al., 2016), the in-scanner computerized tasks were comprised of twelve measures reflecting the same cognitive domains selected from the neuropsychological battery. Details on MRI acquisition are described in Supplementary Material. Each domain included three measures: *reasoning:* Paper Folding (Ekstrom et al., 1976), Matrix Reasoning and Letter Sets; *speed:* Digit Symbol, Letter Comparison (Salthouse & Babcock, 1991), and Pattern Comparison tasks; *memory:* Logical Memory, Word Order Recognition, and Paired Associates; *vocabulary*: Synonyms, Antonyms, and Picture Naming.

### Leisure activities

**Figure 1.**
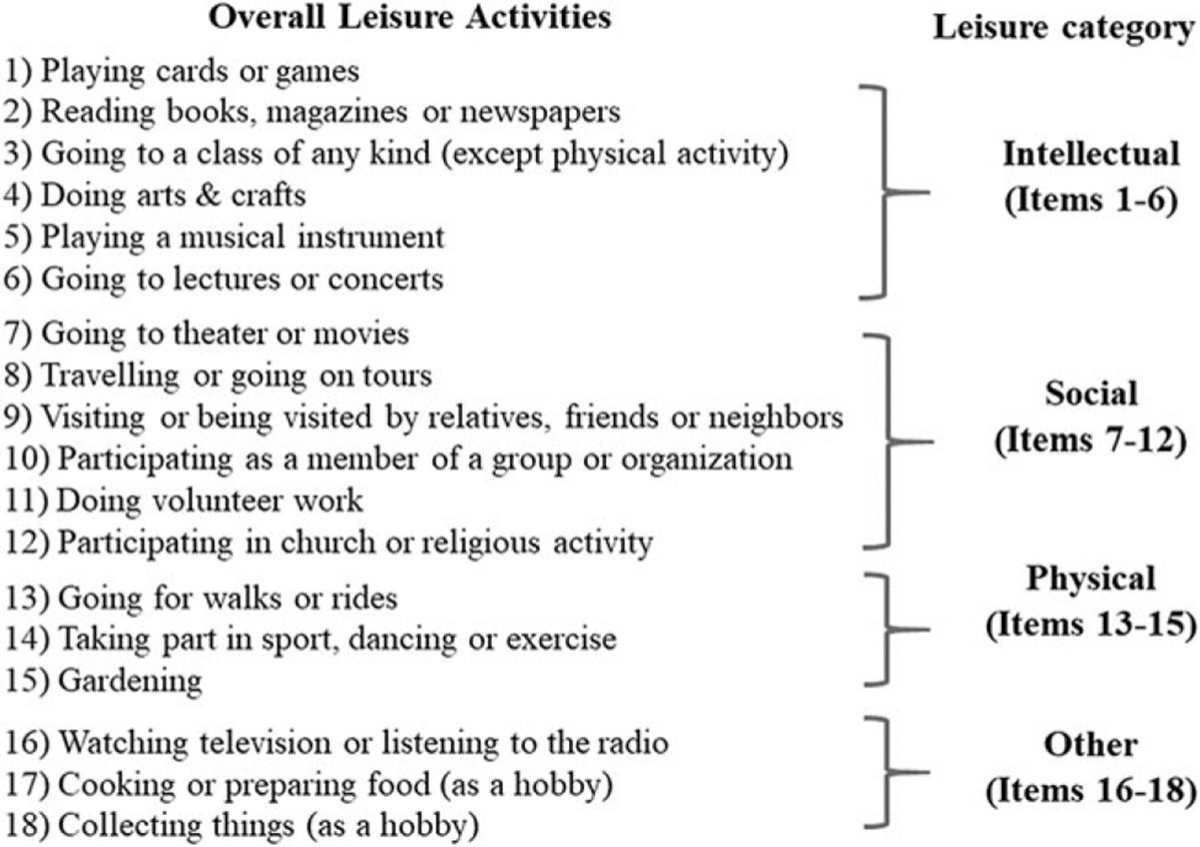
List of leisure activities included in the questionnaire.

The LA questionnaire is an updated version of the 13-item questionnaire previously created and used by our group and collaborators (Armstrong et al., 2021; Helzner et al., 2007; Scarmeas et al., 2001). The questionnaire updated some of the items and added additional activities (gardening, going to lecture or concert, cooking, collecting, art & craft). Participation in the 18 LAs during the previous 6 months was collected at baseline (Figure 1). Participants reported the frequency of their participation in each activity using a 3-point scale: never, sometimes, or often (coded as 0, 1, and 2, respectively). An aggregate summed score (range 0–36) was assigned to each participant reflecting the frequency of leisure engagement. We do not have data on the intensity of each activity.

In secondary analyses, we examined the frequency of LA reflecting three types of activities: cognitive-intellectual, social, and physical. Following previous literature (Armstrong et al., 2021; Helzner et al., 2007; Scarmeas et al., 2001; Verghese et al., 2003), the LA domains were defined by three summed scores of similarly grouped LA items (Figure 1). Intellectual LA was defined by six items (range 0–12) that reflect intellectually demanding activities and activities that engage cognition through art or music. Art and music activities have been considered cognitively demanding at a similar level to other typical intellectual LAs (Salthouse, 2006) and have specific effects on cognitive performance (Verghese et al., 2003; Yu et al., 2020). Social LA was defined by six items (range 0–12) including activities that typically involve some level of social interaction. Physical LA was defined by three items (range 0–6) that reflect activities with evident physical engagement under different effort levels and energy expenditure, which included both physical activity and exercise as defined previously (e.g., going for a walks and rides, gardening, engaging in sports, dance, or exercise).

A confirmatory factor analysis (CFA) was conducted to evaluate the factor structure of LA domains specified a priori (i.e., cognitive-intellectual, social, and physical). Goodness-of-fit measures were based on three model-fit statistics: root mean squared error of approximation (RMSEA) (Steiger, 1990), Tucker–Lewis index (TLI) (Tucker & Lewis, 1973), and comparative fit index (CFI) (Bentler, 1990). General good model-data fit is observed if RMSEA values are lower than 0.06 and if TLI and CFI are higher than 0.95 (Hu & Bender, 1999), in addition, it is acceptable if higher than 0.90 (Bender & Bonett, 1980).

### Covariates

The covariates adopted in the main model were age, gender, years of education, annual family income and cognitive performance at baseline. SES was measured based on self-reported family income in the past 12 months. We treated SES it as a dichotomous variable with ≥$75,000 as reference, based on a median split, since 53% of the sample reported receiving up to $74,999 yearly.

### Statistical Analyses

#### Data description

Demographics, cognitive performance, and LA engagement profile of the participants were described using means and standard deviation. Frequency and percentage were utilized for categorical variables. Characteristics of participants by LA frequency tertiles were compared using analyses of variance (ANOVA) for continuous variables and Pearson’s chi-square for categorical variables. Pearson correlations and t-tests were conducted to examine bivariate relationships among LA and demographics.

#### Latent change score model

**Figure 2.**
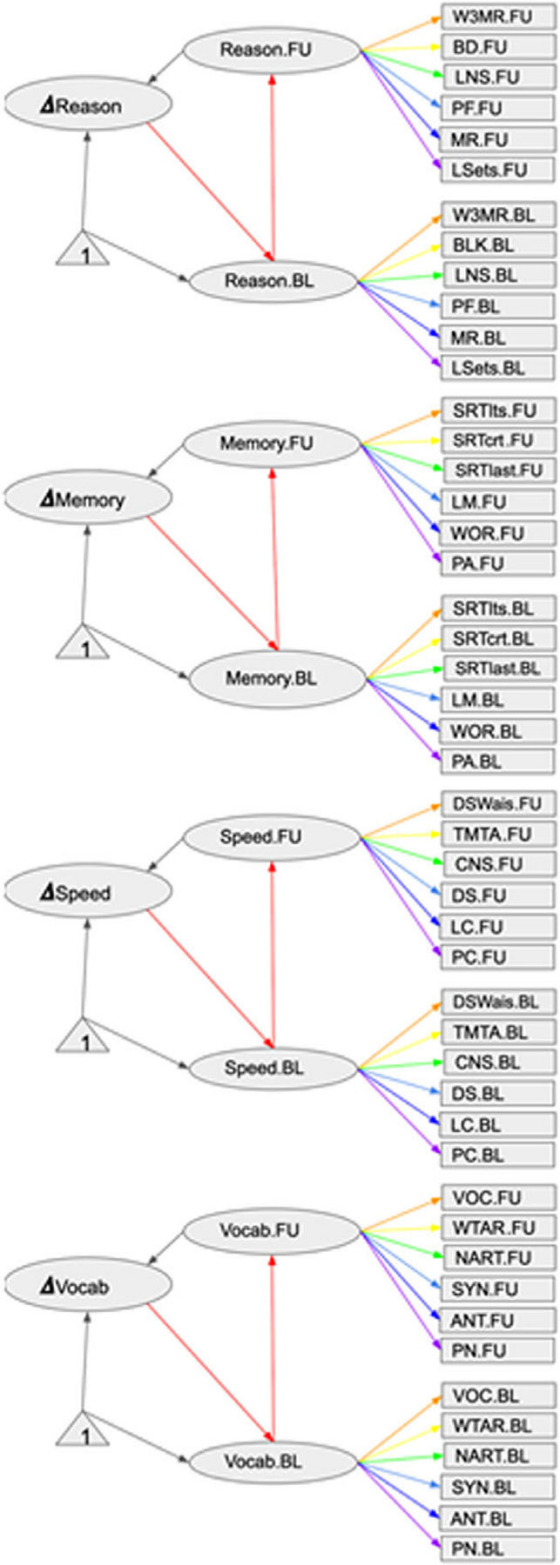
Diagram for the latent change score model. Legend: Coefficients linking indicators and the latent variables at both time points are constrained to be same. Acronyms of the figures are listed by reference abilities: Fluid Reasoning: In scanner-Paper Folding (PF), Matrix Reasoning (MR), Letter Sets (LSets); Out-scanner-Wechsler Adult Intelligence Scale (WAIS-III) Block design task (BD), WAIS III Letter–Number Sequencing test (LNSD), and WAIS III Matrix Reasoning test (W3MR); Processing Speed: In scanner-Digit Symbol (DS), Letter Comparison (LC), Pattern Comparison (PC); Out-scanner- Digit Symbol subtest from the Wechsler Adult Intelligence Scale-Revised (DSWAIS), Part A of the Trail making test (TMTA), and Color naming component of the Stroop (CNS); Memory: In scanner-Logical Memory (LM), Word Order recognition (WOR), Paired Associates (PA); Out-scanner- Selective Reminding Task - long-term storage sub-score (SRTlts), Selective Reminding Task - continuous long-term retrieval (SRTctrl), and Selective Reminding Task - the number of words recalled on the last trial (SRTlast); Vocabulary: In scanner-Synonyms (SYN), Antonyms (ANT), Picture Naming (PN); Out-scanner- vocabulary subtest from the WAIS III (VOC), the Wechsler Test of Adult Reading (WTAR), and American National Adult Reading Test (NART).

We used a multiple indicator latent change score model (LCSM) (Kievit et al., 2018) to generate cognitive scores at baseline and follow-up, as shown in Figure 2 (Gazes et al., 2021), but without covariates. For additional details on the coefficients associated with each of the latent variables, see Table 1 on Supplementary material. The LCSM aims to model change in the latent score rather than observed scores. We modeled the changes of cognitive measures representing the four cognitive domains detailed above, each based on three out-of-scanner and three in-scanner tests, using a traditional confirmatory factor analysis as described in our previous studies (Salthouse et al., 2015). Factor loadings were constrained so that baseline and follow-up loadings were the same. Cognitive change values were calculated as follow-up scores minus baseline scores resulted from LCSM. Positive values indicate increase of cognitive performance and negative values indicate decline in performance over time. We also established the measurement invariance across two time points. The goodness of the model fit was assessed using the CFI, TLI, and RMSEA, with evidence of adequate fit indicated by CFI/TLI ≥ 0.90 and RMSEA ≤ 0.08 (Marsh et al., 2005). The overall fit statistics were close to acceptable range: CFI = 0.85, TLI = 0.84, and RMSEA = 0.069 (95% CI = 0.065–0.072, *p* < 0.001). The full result of the LCSM and its R code is found in Supplementary Material.

#### Association between baseline LA and cognitive change

To examine the relationship between LA and cognitive change, hierarchical multiple regression models were computed using the cognitive change score in each of the cognitive domains as dependent variables and LA as the independent variable, both for total LA frequency in the primary analyses and for each of the LA categories (i.e., cognitive-intellectual, social, and physical) in the secondary analyses. First, the models were adjusted for baseline age only (Model 1), and subsequently adjusted for gender, years of education, SES, and baseline cognitive performance (Model 2). The role of gender as a potential moderator was investigated in Model 3, which included the same covariates as Model 2, as well as the interaction between baseline LA and gender. Similarly, age as a potential moderator was investigated in Model 4, which included the same covariates as Model 2, but also included the interaction between baseline LA and age. Linear moderation effect was investigated using age as a continuous variable (Model 4A), and nonlinear moderation effect was examined using age as categorical variable (Model 4B) considering the three age groups (i.e., young = 21–39 years, middle aged = 40–59 years, and older adults = 60–80 years).

Given that LA participation may influence not only cognitive functioning but brain health measures (Casaletto et al., 2020), in a subset of participants that had available brain measurements (*N* = 140), we added change in thickness to the model, considering all covariates included in our main model (Model 2). By controlling for cortical thickness, we may observe the effect of LA on cognitive change beyond differential brain change (i.e., brain maintenance), which would suggest a cognitive reserve mechanism.

It is relevant to note that we performed a regression with the estimated factor scores as the primary analysis for two reasons. First, the factors were estimated in the larger sample size (*n* = 254) and second, LCSM results in different factor loadings across different models. However, since the LCSM possibly has better power, we also performed a separate LCSM analysis as a supplementary.

Analyses were performed using R package and SPSS 26, and the significance level was set at 0.05. We also provided effect sizes (Cohen’s f^2^) for each regression model, which are interpreted according to Cohen’s guidelines: f^2^ ≥ 0.02 (small), f^2^ ≥ 0.15 (medium), and f^2^ ≥ 0.35 (large) (Cohen, 1988).

## Results

### Sample characteristics

Sample characteristics are described in Table 1. The mean age was 53.8 years (SD = 16.4, range 21–80 years), 56.4% were female, 62.2% White, 24.9% African American, 3.9% Asian, and 11.9% Hispanic. In addition, mean years of education was 16.3 years (SD = 2.3) and approximately half of the participants (53.4%) reported annual family income below <$75,000. Regarding the LA profile, the baseline number of LA was 12.9 activities (SD = 2.3, range 7–18) and the frequency was 19.7 (SD = 4.4, range 8.4–32). Older individuals reported greater engagement in LA (*p* = .04), and, at a trend level, individuals with higher education reported higher LA engagement (*p* = .05). Although there was no gender effect on the overall LA frequency, cooking as a LA was significantly higher in women (*p* = .007).

Correlation analysis (Table 2, Supplementary Material) revealed that age was positively associated with overall LA frequency (*p* = .02) and negatively associated with baseline performance, indicating that reasoning, memory, and speed domains decreased as a function of age, whereas vocabulary increased with aging (*ps.* < .001). Age was also negatively associated with cognitive change scores, indicating a steeper decline in older individuals (*ps.* < .001), however the association was not significant for memory. Education was positively associated with LA frequency (*p* = .01), and baseline performance for reasoning, vocabulary (*ps.* < .001) and memory (*p* = .008), but negatively associated with change in vocabulary (*p* = .04), which did not remain significant after controlling for age (*p* = .16). T-tests revealed an effect of gender on vocabulary baseline scores, indicating women perform better (*p* = .01), and SES was associated with baseline performance for vocabulary and reasoning (*ps.* = .001), indicating poorer performance in those with lower annual income.

### Relationship between LA frequency and cognitive change

**Figure 3.**
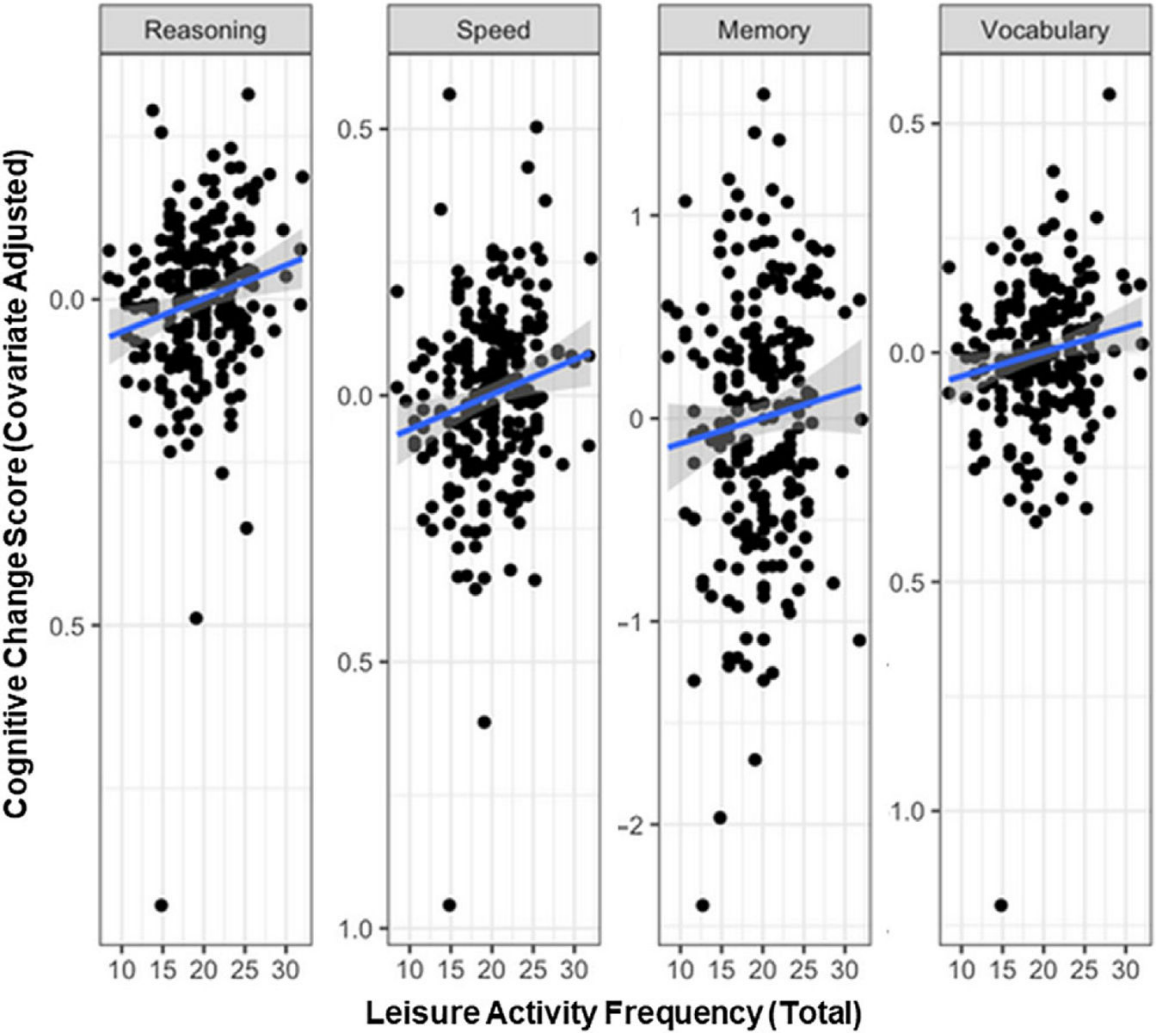
Associations between total leisure activity frequency and cognitive change.

In Model 1 (adjusted only for age) we observed that higher baseline LA frequency was associated with lower rates of cognitive decline for reasoning (*p* = .003) and speed (*p* = .004) (Table 2). In Model 2 (adjusted for age and demographics), we observed that higher LA frequency was associated with improvement on vocabulary (*p* = .02) and lower rates of cognitive decline for reasoning (*p* = .003), speed (*p* = .004), and at trend level, for memory (*p* = .05) (Table 2, Figure 3). A similar pattern was observed in a model not including SES as a covariate, since there was missing data for 24 participants (Table 3, Supplementary Material). It is worth mentioning that in the main model (Model 2), the effect sizes (f^2^) ranged from medium (memory and vocabulary) to large (speed and reasoning), and most results survived the Bonferroni correction, except memory, as indicted in Table 2. Our results remained similar when analyzing the data using LSCM approach (Supplementary Material, R code link).

The associations between each LA category and cognitive change (Table 3) followed a similar pattern observed in the main finding (Model 2). For instance, higher social-LA frequency predicted vocabulary improvement (at a trend level) and slower rates of cognitive decline for reasoning and speed. Similarly, higher cognitive-intellectual LA was associated with slower decline of reasoning and memory, and at trend level for speed. No associations were observed between cognitive change and physical LA.

### Confirmatory factor analysis

The CFA indicated an acceptable fit for the three-factor model of LA including cognitive-intellectual, social, and physical domains. The RMSEA of 0.058 indicates a good fit (lower than 0.06), and CFI of 0.86 and TLI of 0.83 are marginal but close to the acceptable fit of 0.90. The detailed CFA diagram is presented as Supplementary Material (Figure 1). Since the model fit was marginal, we ran another CFA including only the items with significant loadings, which resulted in a better fit (CFI of 0.90 and TLI of 0.87 – Figure 2, Supplementary Material) and did not significantly alter the LA-cognitive change associations (Table 4, Supplementary Material).

### Test for brain maintenance effect

Even though LA was not associated with the thickness change (*p* > .05), individual differences in structural change might influence cognitive changes. Therefore, in a subset of participants (*N* = 140), we tested how change in thickness influenced the effects of LA on cognitive change, considering the same covariates included in Model 2. Total-LA predicted lower rates of cognitive decline for reasoning (*p* = .02) and speed (*p* = .03), but not for memory and vocabulary (Table 5, Supplementary Material).

### Gender moderation

Gender moderated the association between baseline LA frequency and cognitive change for reasoning (*p* = .02) and speed (*p* = .03) (Table 4). The analyses stratified by gender revealed that these associations occurred only in men (*ps* = .001) and was not significant for women (*ps* > .05).

### Age moderation

Age did not moderate the association between baseline LA engagement and cognitive change across all cognitive domains (Table 6, Supplementary Material).

## Discussion

The overall study aim was to examine the relationship between self-reported measures of LA frequency and cognitive change over a span of 5 years across adulthood. We found that greater baseline LA engagement relates to less decline across cognitive domains (i.e., reasoning, speed, and episodic memory), and better vocabulary performance. Both social and cognitive-intellectual LA predicted less cognitive decline in reasoning, however LA components presented specific associations with cognitive change: social-LA was associated with less decline on speed and better vocabulary, and cognitive-intellectual-LA was associated with less memory decline. Physical-LA was not associated with cognitive change. Additionally, the relationships between LA frequency and cognitive change were shown to be stable across adulthood since age did not moderate these associations. Moreover, our data revealed gender as a moderator of LA-cognition associations, indicating stronger association in men.

Our results agree with previous longitudinal studies examining the association between baseline LA and cognitive change in healthy middle-aged individuals and older adults (Ghisletta et al., 2006; Lovden et al., 2005; Richards et al., 2003; Small et al., 2012; Wang et al., 2013; Yates et al., 2016). Critically, LA protective benefits have been observed in several cognitive domains, such as global cognition, language, executive functions (Wang et al., 2013; Yates et al., 2016), speed processing (Ghisletta et al., 2006; Lovden et al., 2005; Small et al., 2012; Yates et al., 2016) and episodic memory (Richards et al., 2003; Small et al., 2012; Wang et al., 2013; Yates et al., 2016). A novel finding from our study is that LA was not only associated with less cognitive decline but also with better performance on vocabulary or crystallized intelligence, as this domain has been shown to improve over time (Gazes et al., 2021; Salthouse, 1998, 2010). Interestingly, our finding was consistent across these cognitive domains with effect sizes ranging from medium (vocabulary) to large (reasoning and speed). Our findings support the cognitive reserve framework, in line with the differential preservation hypothesis (Salthouse, 2006), stating that above and beyond several demographic characteristics and differential brain change, an active lifestyle confers protective effects on cognitive functioning and helps individuals to cope better with aging.

Our results contrast with previous findings that LA was associated with cognitive ability but not change over time (Bielak et al., 2012; Gow et al., 2014; Gow et al., 2017; Mitchell et al., 2012). These studies support the preserved differentiation hypothesis, which states that people who are more mentally active tend to have high levels of cognitive functioning throughout their lives, and thus differences in performance are preserved across adulthood (Salthouse, 2006). While it remains inconclusive if LA level affects subsequent cognitive change, and/or whether cognitive ability level at baseline predicts LA participation (Bielak, 2010; Gow et al., 2014), our results support the former owing to the fact that we controlled for baseline cognitive ability, and intervention studies suggest the same (Kivipelto et al., 2018; Leanos et al., 2020; Park et al., 2014). Nevertheless, we cannot rule out that premorbid intelligence or any subtle cognitive difficulty may have influenced LA engagement.

We observed a consistent cognitive benefit of total LA frequency, a variable reflecting a variety of activities. This is in line with the reported benefits of “activity diversity” (Lee et al., 2020) and learning multiple skills simultaneously (Leanos et al., 2020). When examining the effects of different components separately, social and cognitive-intellectual LAs were those that drove the main findings: both LA components benefit reasoning trajectory, however social LA seemed more relevant for speed and vocabulary trajectory, and cognitive-intellectual LA for memory trajectory. It is possible that these differences occurred by chance or may have been influenced by dose or level of effort of the LAs. In addition, the division between social and cognitive domains might be questionable, since these modalities may be stimulated simultaneously (e.g., playing cards/games, playing musical instruments, going to classes) and therefore provide synergetic effects.

Although the specific mechanism through which LA influences cognitive functioning remains unclear, intellectual enrichment has been associated with increased synaptic density in mice (Cracchiolo et al., 2007), while in humans, cognitive activity/training is associated with brain structural and functional benefits, supporting its role in maintaining better brain health. For instance, cognitive training has been associated with increased white matter (Lovden et al., 2010), grey matter and cortical volume (Nguyen et al., 2019) and better network efficiency and plasticity of neural circuits (Cheng, 2016; Miotto et al., 2018). Similarly, social activity engagement can also provide mental stimulation through complex communication and interaction with others (Bennett et al., 2006; Evans et al., 2019; Fratiglioni et al., 2020), but also benefit through other mechanisms such as stress management and emotional support.

Surprisingly, we observed no association between physical LA and cognitive change, in contrast with the robust literature on the benefits of physical activity on cognitive/brain functioning and reduced cardiovascular disease risk (Fratiglioni et al., 2020; Ogino et al., 2019). There are several reasons why we were unable to find a relationship between physical LA and cognitive change: (1) the reduced number of questions addressing physical activity in our questionnaire (three items); (2) the self-report nature of the data; and (3) the lack of specificity on the activity, such as duration, intensity, and attendance.

It is relevant to highlight that the LA domains were chosen based on similar structures observed in previous works (Armstrong et al., 2021; Helzner et al., 2007; Scarmeas et al., 2001). Critically, our CFA model presented an acceptable fit for the 3-factor model. For instance, the RMSEA value observed in the model is in line with the recommendation of a good model fit of RMSEA < 0.06. The CFI value and TLI were a little below the criteria of 0.95, but slightly close to the acceptable fit of 0.09 (Bentler & Bonett, 1980). Based on these CFA indices, we consider this sample as an acceptable fit to the 3-factor model. In addition, when we optimize our model fit selecting only the items with significant loadings, the results remained similar.

Another novel contribution of our study is the evidence of gender moderation on reasoning and speed, indicating that the protective role of LA on cognition was stronger in men (for both domains). In our sample, we did not find gender differences in overall LA frequency, so the explanation of our findings remains unclear. Our findings suggest that men and women may engage differently in LA, which may also modify its potential protective role to age-related cognitive decline. This is in line with a few studies that observed gender differences in LA engagement and its association with cognitive performance and trajectories (Carlson et al., 2008; Crowe et al., 2003; Hassing, 2020). Future research should better address the gender differences and develop interventions to address potential gaps. We cannot rule out the possibility that our finding is just a reflection of our sample characteristics.

Our findings did not support the prediction that the association between LA and cognitive change would be stronger at older age (Bielak et al., 2012; Bielak, 2010; Bielak et al., 2014; Hillman et al., 2006; Ogino et al., 2019). In contrast, our results are in line with studies that did not observe a greater effect of cognitive activity on cognition in older adults (Newson & Kemps, 2006; Salthouse et al., 2002). The absence of age moderation suggests that LA engagement and an active lifestyle are relevant for preserving cognitive functioning in any stage of adult life, and not only at older age. We do not rule out the possibility that sample size or characteristics and cohort effects may have influenced our findings. Our results support the development of prevention-focused intervention to younger and older populations. In addition, it is possible that LA engagement is linked to, reflects or influences other relevant clinical outcomes, such as general health and mood/depression (Sharifian et al., 2020).

The strengths of our study that enhances LA research include (1) a wide age range, addressing LA across adulthood; (2) an aggregate LA frequency score comprising of a range of common everyday life activities, and concomitantly addressing specific LA components defined statistically and based on previous works; (3) a robust measurement of cognitive abilities well established to change with aging (Habeck et al., 2016; Salthouse et al., 2015) comprising of multiple cognitive measures (six within each cognitive domain); (4) the use of latent change score modeling to identify latent cognitive variables at baseline and follow-up under similar parameters, which allowed us to model within and between person variances; (5) models that comprise critical demographics beyond age and gender, such as education and SES; (6) exploratory analysis on the effect of brain measures on the effect of LA on cognitive performance; and (7) moderation analysis of gender and age, both aspects with limited evidence.

The study limitations should also be noted. First, generalizability is limited since the sample is relatively modest in size, highly educated, and majority white (especially the older individuals). Future research needs samples adequately powered to investigate the roles of education, gender, and race/ethnicity in these associations. An obvious limitation inherent in the design is the self-report nature of the data, which may be influenced by recall bias and have reduced accuracy. Another limitation is the lack of data on retirement/work status and general health, which likely influences available time to engage in LA. Although we adopted a longitudinal design, we did not address the potential bidirectional association between LA and cognition, and therefore, reverse causality still is a confounder.

In conclusion, our results expand on previous literature by showing a positive association between active lifestyle and positive cognitive trajectories in a well characterized, cognitively heathy sample across adulthood. Initial LA frequency benefits cognitive trajectories over 5 years, above and beyond demographics and level of cognitive ability. Its potential protective effects seem to occur similarly across adulthood, supporting a life-course approach. Gender differences were shown to be a relevant factor that modifies the LA-cognitive change association. Our results suggest that LA is a proxy for cognitive reserve and serves as a potential buffer against age-related cognitive decline across adulthood and genders, with implications for future reserve-enhancing interventions and prevention trials, which may be needed to begin before old age.

## Supplementary Material

Supplemental Material

## Figures and Tables

**Table 1.** Demographic and cognitive characteristics of the study participants by tertiles of leisure activity frequency

Variables	Overall sample (*N* = 234)	Total LA frequency, lower tertile (*N* = 75)	Total LA frequency, middle tertile (*N* = 80)	Total LA frequency, higher tertile (*N* = 79)	*p*-value^a^
Age, mean (SD) [range 21–80 YO]	53.8 (16.8)	52.1 (17.5)	51.6 (16.4)	57.5 (16.2)	.04*
Age Groups					
Young (21–39 YO) *N* (%)	59 (25.2%)	22 (37.3%)	22 (37.3%)	15 (25.4%)	.28
Middle Age (40–59 YO) *N* (%)	53 (22.6%)	17 (32.1%)	21 (39.6%)	15 (28.3%)	
Older (60–80 YO) *N* (%)	122 (52.1%)	36 (29.5%)	37 (30.3%)	49 (40.2%)	
Female *N* (%)	132 (56.4 %)	38 (50.7 %)	44 (55.0%)	50 (63.3 %)	.27
Race/Ethnicity					
White *N* (%)	145 (62.2%)	46 (31.7%)	46 (31.7%)	53 (36.6%)	.85
African American *N* (%)	58 (24.9%)	19 (32.8%)	23 (39.7%)	16 (27.6%)	
Asian *N* (%)	9 (3.9%)	2 (22.2%)	3 (33.3%)	4 (44.4%)	
Hispanics *N* (%)	28 (12.0%)	7 (25.0%)	14 (50.0%)	7 (25.0%)	.17
Education (years), mean (SD)	16.3 (2.3)	15.8 (2.3)	16.5 (2.5)	16.6 (2.2)	.05
AMNART IQ, mean (SD)	117.7 (8.0)	116.6 (7.3)	117.2 (8.6)	119.3 (7.9)	.09
Annual Family Income < $75.000 *N* (%)^b^	111 (53.4%)	33 (53.2%)	41 (54.7%)	37 (52.1%)	.23
Reasoning BL score, mean (SD) [range −1.6, 1.7]	.001 (0.7)	.004 (0.7)	.041 (0.7)	−.042 (0.7)	.36
Speed BL score, mean (SD) [range −1.7, 2.1]	.012 (0.7)	.067 (0.7)	.052 (0.7)	−.080 (0.6)	.77
Memory BL score, mean (SD) [range −2.2, 1.7]	.017 (0.8)	.059 (0.8)	.039 (0.8)	−.044 (0.8)	.72
Vocabulary BL score, mean (SD) [range −2.3, 1.0]	.001 (0.7)	−.116 (0.7)	−.014 (0.8)	.128 (0.7)	.13
Reasoning CH score, mean (SD) [range −1.1, 0.1]	−.150 (0.1)	−.164 (0.1)	−.142 (0.1)	−.143 (0.1)	.54
Speed CH score, mean (SD) [range −1.2, 0.4]	−.178 (0.1)	−.195 (0.2)	−.177 (0.1)	−.164 (0.1)	.57
Memory CH score, mean (SD) [range −2.5, 1.4]	−.126 (0.6)	−.193 (0.7)	−.144 (0.7)	−.045 (0.5)	.37
Vocabulary CH score, mean (SD) [range −1.3, 0.6]	.065 (0.1)	.053 (0.2)	.082 (0.1)	.059 (0.1)	.58
Frequency of LA, mean (SD) [range 8.4, 32]	19.7 (4.4)	–	–	–	–
Intellectual LA, mean (SD) [range 2, 11.7]	6.6 (1.9)	–	–	–	–
Social LA, mean (SD) [range 1, 12]	6.3 (2.2)	–	–	–	–
Physical LA, mean (SD) [range 0, 6]	3.2 (1.1)	–	–	–	–

BL: baseline; CH: change (follow-up minus baseline); LA: leisure activity; SD: standard deviation; YO: years old.

a *p* values from ANOVA for continuous variables and chi-squared for categorical variables.

b Missing data for 26 participants.

* *p*-values <.05.

**Table 2.** Association between leisure activity frequency at baseline and cognitive change

	Reasoning change (*N* = 234)		Speed change (*N* = 234)		Memory change (*N* = 234)		Vocabulary change (*N* = 234)	
	R^2^ = .21; f^2^ = .26; F(2231) = 32.357^a^		R^2^ = .17; f^2^ = .20; F(2231) = 24.874^a^		R^2^ = .02; f^2^ = .02; F(2231) = 2.512^b^		R^2^ = .14; f^2^ = .16; F(2231) = 19.249^a^	
Model 1	ß	*p*-value	ß	*p*-value	ß	*p*-value	ß	*p*-value
Age	−.460	<.001*	−.409	<.001*	−.103	.11	−.373	<.001*
Baseline LA	.176	**.003** *	.173	**.004** *	.119	.07	.103	.09

	Reasoning change (*N* = 208)		Speed change (*N* = 208)		Memory change (*N* = 208)		Vocabulary change (*N* = 208)	
	R^2^ = .24; f^2^ = .31; F(6201) = 10.993^a^		R^2^ = .19; f^2^ = .23; F(6201) = 8.249^a^		R^2^ = .12; f^2^ = .13; F(6201) = 4.810^b^		R^2^ = .16; f^2^ = .19; F(6201) = 6.575^a^	
Model 2	ß	*p*-value	*p*-value	*p*-value	ß	*p*-value		
Age	−.423	<.001*	−.359	<.001*	−.255	.001*	−.348	<.001*
Gender	−.023	.70	−.044	.49	−.066	.32	−.027	.68
Education	.125	.08	.135	.04*	.090	.21	−.028	.70
family income	.065	.30	.073	.26	−.005	.94	−.008	.90
Baseline performance	.073	.35	.059	.47	−.363	<.001*	−.130	.07
Baseline LA	.195	**.002** *	.190	**.004** *	.132	.05*	.152	**.02** *

LA: leisure activity; ß: standardized regression coefficient; gender: F= 0, M = 1; family income: <75.000 = 0. >75.000 = 1; didn’t know = 2 (10 participants). Results in each model that survived Bonferroni correction are highlighted in bold.

a *p* < .0001.

b Non-significant model.

* *p*-values ≤ .05.

**Table 3.** Association between categories of leisure activity frequency at baseline and cognitive change

	Reasoning change (*N* = 208)		Speed change (*N* = 208)		Memory change (*N* = 208)		Vocabulary change (*N* = 208)	
	R^2^ = .22; f^2^ = .28; F(6201) = 9.942^a^		R^2^ = .17; f^2^ = .20; F(6201) = 7.292^a^		R^2^ = .12; f^2^ = .13; F(6201) = 4.980^a^		R^2^ = .15; f^2^ = .17; F(6201) = 6.020^a^	
Variables	ß	*p*-value	ß	*p*-value	ß	*p*-value	ß	*p*-value
Age	−.415	<.001	−.351	<.001	−.257	<.001	−.334	<.001
Gender	−.026	.68	−.048	.45	−.066	.32	−.029	.65
Education	.135	.06	.141	.03	.087	.22	−.022	.76
Family income	.070	.27	.076	.25	.005	.94	.010	.88
Baseline performance	.055	.49	.046	.59	−383	<.001	−.135	.07
Intellect LA	.139	.03*	.128	.05	.147	.03*	.104	.12
	Reasoning change (*N* = 208)		Speed change (*N* = 208)		Memory change (*N* = 208)		Vocabulary change (*N* = 208)	
	R^2^ = .23; f^2^ = .29; F(6201) = 10.122^a^		R^2^ = .18; f^2^ = .21; F(6201) = 7.519^a^		R^2^ = .11; f^2^ = .12; F(6201) = 4.317^a^		R^2^ = .15; f^2^ = .17; F(6201) = 6.298^a^	
Variables	ß	*p*-value	ß	*p*-value	ß	*p*-value	ß	*p*-value
Age	−.401	<.001*	−.339	<.001*	−.244	.002*	−.346	<.001*
Gender	−.034	.58	−.053	.41	−.073	.27	−.037	.57
Education	.120	.09	.138	.04*	.098	.18	−.033	.65
Family income	.052	.41	.062	.34	−.012	.86	−.000	.99
Baseline performance	.098	.22	.078	.35	−.362	<.001*	−.112	.12
Social LA	.149	.02*	.145	.02*	.074	.28	.129	.05*
	Reasoning change (*N* = 208)		Speed change (*N* = 208)		Memory change (*N* = 208)		Vocabulary change (*N* = 208)	
	R^2^ = .21; f^2^ = .26; F(6201) = 9,196^a^		R^2^ = .16; f^2^ = .19; F(6201) = 6.695^a^		R^2^ = .11; f^2^ = .12; F(6201) = 4.220^b^		R^2^ = .14; f^2^ = .16; F(6201) = 5.609^a^	
Variables	ß	*p*-value	ß	*p*-value	ß	*p*-value	ß	*p*-value
Age	−.395	<.001*	−.333	<.001*	−.239	.002*	−.327	<.001*
Gender	−.040	.52	−.060	.36	−.078	.24	−.040	.54
Education	.152	.03*	.163	.01*	.110	.12	−.008	.91
Family income	.049	.45	.058	.38	−.015	.82	−.004	.94
Baseline performance	.074	.36	.059	.48	−.370	<.001*	−.123	.106
Physical LA	.067	.29	.056	.38	.053	.42	.031	.64

LA: leisure activity; ß: standardized regression coefficient; gender: F = 0, M = 1; family income: <75.000 = 0. >75.000= 1; didn’t know = 2 (10 participants).

a *p* < .0001.

b *p* = .001.

* *p*-values ≤ .05.

**Table 4.** Gender moderation of the association between leisure activity and cognitive change

	Reasoning change (*N* = 208)		Speed change (*N* = 208)		Memory change (*N* = 208)		Vocabulary change (*N* = 208)	
	R2 = .25; f^2^ = .33; F(7200) = 10.397^a^		R2 = .21; f^2^ = .26; F(7200) = 7.887^a^		R2 = .12; f^2^ = .13; F(7200) = 4.136^a^		R2 = .16; f^2^ = .19; F(7200) = 6.060^a^	
Model 3	ß	*p*-value	ß	*p*-value	ß	*p*-value	ß	*p*-value
Age	−.419	<.001	−.358	<.001	−.254	.001*	−.350	<.001*
Gender	−.681	.02*	−.684	.02*	−.207	0.51	−.515	0.09
Education	0.134	0.05	0.148	.02*	0.092	0.2	−.017	0.81
Family income	0.058	0.35	0.067	0.3	−.006	0.93	0.004	0.95
Baseline performance	0.084	0.28	0.065	0.42	−.360	<.001*	−.131	0.07
Baseline LA	0.058	0.59	0.056	0.52	0.103	0.27	0.05	0.58
Baseline LA × gender	0.679	.02*	0.661	.03*	0.146	0.64	0.504	0.105

LA: leisure activity; ß: standardized regression coefficient; gender: F = 0, M = 1; family income: <75.000 = 0. >75.000 = 1; didn’t know = 2 (10 participants).

a *p* < 0.0001.

* *p*-values ≤ .05.
