# Supplementary material for: Leisure activity engagement across adulthood predicts cognitive change after five years: Do gender and age matter?
Source: J Int Neuropsychol Soc. Author manuscript; Available in PMC 2023 Jul 10. (PMC10186268; doi:10.1017/S1355617722000510)

**Supplemental Material**

**Description**

1. **Image acquisition and processing (Gazes et al., 2021)**

All MR images were acquired on a 3.0T Philips Achieva Magnet. There were two 2-hour MR imaging sessions to accommodate the twelve fMRI tasks as well as the additional imaging modalities. Relevant to the current study, T1-weighted MPRAGE scan was acquired to determine cortical thickness, with a TE/TR of 3/6.5 ms and Flip Angle of 8°, in-plane resolution of 256 x 256, field of view of 25.4 × 25.4cm, and 165–180 slices in axial direction with slice-thickness/gap of 1/0mm. In addition, BOLD fMRI for twelve tasks, FLAIR, DTI, ASL and a 7-minute resting BOLD scan were acquired but not reported in the current study. A neuroradiologist reviewed each subject's scans. Any significant findings were conveyed to the subject's primary care physician.

Each subject's structural T1 scans were reconstructed using FreeSurfer v5.1 (http://surfer.nmr.mgh.harvard.edu/). The accuracy of FreeSurfer's subcortical segmentation and cortical parcellation (Fischl et al., 2002; Fischl et al., 2004) has been reported to be comparable to manual labeling. Each subject's white and gray matter boundaries as well as gray matter and cerebral spinal fluid boundaries were visually inspected slice by slice, manual control points were added when any visible discrepancy was found, and reconstruction was repeated until we reached satisfactory results within every subject. The subcortical structure borders were plotted by FreeView visualization tools and compared against the actual brain regions. In case of discrepancy, they were corrected manually. Finally, we computed the mean cortical thickness for each participant to be used in group-level analyses.

1. **Latent Change Score Model**

The supplementary Table 1 reports the factor loadings of the LCSM that we estimated the factor scores. We note that since we constraint the factor loadings at baseline and follow-up to be same. The full results and its corresponding R codes can be found on <https://seonjoo.github.io/lcsa_leisure>. Of note, the results agree with the regression results.

| Supplemental Table 1  LCSM Factor Loadings | | | | | | |
| --- | --- | --- | --- | --- | --- | --- |
|  | **Variables** |  | **Estimate** | **Std.Err** | **z-value** | **P(>\|z\|)** |
| Speed at baseline | WAISRRAW_bl | (S1) | 1 |  |  |  |
|  | TMTAtm_bl | (S2) | -0.883 | 0.071 | -12.482 | <0.001 |
|  | STRPcRAW_bl | (S3) | 0.925 | 0.066 | 13.999 | <0.001 |
|  | DgtS_CRT_bl | (S4) | -1.155 | 0.075 | -15.339 | <0.001 |
|  | PttC_CRT_bl | (S5) | -0.909 | 0.078 | -11.727 | <0.001 |
|  | LtCm_CRT_bl | (S6) | -0.961 | 0.077 | -12.462 | <0.001 |
| Speed at follow up | WAISRRAW_fu | (S1) | 1 |  |  | <0.001 |
|  | TMTAtim_fu | (S2) | -0.883 | 0.071 | -12.482 | <0.001 |
|  | STRPcRAW_ | (S3) | 0.925 | 0.066 | 13.999 | <0.001 |
|  | DgtS_CRT_ | (S4) | -1.155 | 0.075 | -15.339 | <0.001 |
|  | PttC_CRT_ | (S5) | -0.909 | 0.078 | -11.727 | <0.001 |
|  | LtCm_CRT_ | (S6) | -0.961 | 0.077 | -12.462 | <0.001 |
| Reasoning at baseline | WAIS3RAW_ | (R1) | 1 |  |  | <0.001 |
|  | BLKraw_bl | (R2) | 0.956 | 0.062 | 15.343 | <0.001 |
|  | WAIS3RAW_ | (R3) | 0.703 | 0.063 | 11.153 | <0.001 |
|  | MtR_POTC_ | (R4) | 0.956 | 0.064 | 14.962 | <0.001 |
|  | PpF_POTC_ | (R5) | 0.967 | 0.068 | 14.282 | <0.001 |
|  | LtS_POTC_ | (R6) | 0.925 | 0.072 | 12.931 | <0.001 |
| Reasoning at follow up | WAIS3RAW_ | (R1) | 1 |  |  | <0.001 |
|  | BLKraw_fu | (R2) | 0.956 | 0.062 | 15.343 | <0.001 |
|  | WAIS3RAW_ | (R3) | 0.703 | 0.063 | 11.153 | <0.001 |
|  | MtR_POTC_ | (R4) | 0.956 | 0.064 | 14.962 | <0.001 |
|  | PpF_POTC_ | (R5) | 0.967 | 0.068 | 14.282 | <0.001 |
|  | LtS_POTC_ | (R6) | 0.925 | 0.072 | 12.931 | <0.001 |
| Memory at baseline | SRTlts_bl | (M1) | 1 |  |  | <0.001 |
|  | SRTcltr_b | (M2) | 1.024 | 0.028 | 36.008 | <0.001 |
|  | SRTlst_bl | (M3) | 0.962 | 0.036 | 26.942 | <0.001 |
|  | LgM_POTC_ | (M4) | 0.373 | 0.059 | 6.282 | <0.001 |
|  | PrA_POTC_ | (M5) | 0.372 | 0.06 | 6.152 | <0.001 |
|  | WrO_POTC_ | (M6) | 0.435 | 0.057 | 7.66 | <0.001 |
| Memory at follow up | SRTlts_fu | (M1) | 1 |  |  |  |
|  | SRTcltr_f | (M2) | 1.024 | 0.028 | 36.008 | <0.001 |
|  | SRTlast_f | (M3) | 0.962 | 0.036 | 26.942 | <0.001 |
|  | LgM_POTC_ | (M4) | 0.373 | 0.059 | 6.282 | <0.001 |
|  | PrA_POTC_ | (M5) | 0.372 | 0.06 | 6.152 | <0.001 |
|  | WrO_POTC_ | (M6) | 0.435 | 0.057 | 7.66 | <0.001 |
| Vocab at baseline | WAISRRAW_ | (V1) | 1 |  |  | <0.001 |
|  | WTARrw_bl | (V2) | 1.15 | 0.054 | 21.489 | <0.001 |
|  | AMNARTrr_ | (V3) | -1.098 | 0.055 | -19.981 | <0.001 |
|  | Syn_POTC_ | (V4) | 0.978 | 0.041 | 23.673 | <0.001 |
|  | Ant_POTC_ | (V5) | 1.086 | 0.055 | 19.816 | <0.001 |
|  | PctNm_Pr_ | (V6) | -1.058 | 0.056 | -18.985 | <0.001 |
| Vocab at follow up | Syn_POTC_ | (V1) | 1 |  |  | <0.001 |
|  | Ant_POTC_ | (V2) | 1.15 | 0.054 | 21.489 | <0.001 |
|  | PctNm_Pr_ | (V3) | -1.098 | 0.055 | -19.981 | <0.001 |
|  | WAISRRAW_ | (V4) | 0.978 | 0.041 | 23.673 | <0.001 |
|  | WTARraw_f | (V5) | 1.086 | 0.055 | 19.816 | <0.001 |
|  | AMNARTrr_ | (V6) | -1.058 | 0.056 | -18.985 | <0.001 |

**Supplemental Table 2**

Bi-variate relationships between demographics, leisure activity and cognition

|  | Overall  LA Frequency | Reasoning  Baseline | Speed  Baseline | Memory Baseline | Vocab  Baseline | Reasoning  Change | Speed  Change | Memory  Change | Vocab  Change |
| --- | --- | --- | --- | --- | --- | --- | --- | --- | --- |
| Age | r= .13  p = .03* | r= -.45  p < .001* | r= -.65  p < .001* | r= -.45  p < .001* | r= .18  p = .005* | r= -.43  p < .001* | r= -.38  p < .001* | r= -.08  p = .19 | r= -.35  p < .001* |
| Gender | t = 1.36  p = .17 | t= -.60  p = .54 | t= 1.63  p = .10 | t= 1.53  p = .12 | t= -2.60  p = .010* | t= 1.33  p = .18 | t= 1.72  p = .08 | t= .96  p = .33 | t= 1.50  p = .13 |
| Education | r= .15  p = .01* | r= .33  p < .001* | r= .04  p = .51 | r= .17  p = .008* | r= .41  p < .001* | r= .09  p = .14 | r= .09  p = .16 | r= -.01  p = .84 | r= -.18  p = .005* |
| Family Inc | t= -.64  p = .51 | t= -3.44  p = .001* | t= -.79  p = .42 | t= -.08  p = .93 | t= -3,38  p = .001* | t= -1.11  p = .26 | t= -.85  p = .34 | t= .38  p = .97 | t= 1.00  p = .31 |

*Legend:* LA = Leisure activity; r = Pearson correlations, t = t-tests. Gender : F= 0, M= 1; Family Income: <$75.000 = 0, ≥$75.000 = 1. * represent p-values < .05.

**Supplemental Table 3**

Baseline leisure activity frequency and cognitive change

(Family income not included in the model)

| Model 2 | Reasoning Change  N=234 | | Speed Change  N=234 | | Memory Change  N=234 | | Vocabulary Change  N=234 | |
| --- | --- | --- | --- | --- | --- | --- | --- | --- |
|  | R^2^= .24  F(5,228)= 14.973^a^ | | R^2^= .20  F(5,228)= 11.391^a^ | | R^2^=.12  F(5,228)= 4.815^a^ | | R^2^= .16  F(5,228)= 8.923^a^ | |
|  | ß | p-value | ß | p-value | ß | p-value | ß | p-value |
| Age | -.414 | <.001* | -.363 | <.001* | -.274 | .001* | -.342 | <.001* |
| Gender | -.044 | .45 | -.064 | .29 | -.071 | .23 | -.032 | .60 |
| Education | .091 | .16 | .118 | .05* | .078 | .26 | -.050 | .46 |
| Baseline performance | .110 | .12 | .079 | .31 | -.374 | <.001* | -.128 | .06 |
| Baseline LA | .154 | .01* | .148 | .01* | .09 | .12 | .118 | .05* |

*Legend:* LA: Leisure Activity; ß: standardized regression coefficient; ^a^*p* < 0.001; Gender: F = 0, M = 1; Family Income: <$75.000 = 0, ≥$75.000 = 1; didn’t know = 2 (10 participants). * p-values ≤ .05.

**Supplemental Table 4**

Association between categories of leisure activity frequency at baseline and cognitive change (Based on CFA represented on Supplemental Figure 2)

|  | Reasoning Change  N=208 | | Speed Change  N=208 | | Memory Change  N=208 | | Vocabulary Change  N=208 | |
| --- | --- | --- | --- | --- | --- | --- | --- | --- |
| Variables | R^2^= .22 f^2^=.28  F(6,201)= 9.912** | | R^2^= .17 f^2^=.20  F(6,201)= 7.255** | | R^2^=.12 f^2^=.13  F(6,201)= 5.100** | | R^2^= .15 f^2^=.17  F(6,201)= 6.150** | |
|  | ß | p-value | ß | p-value | ß | p-value | ß | p-value |
| Age | -.406 | <.001 | -.339 | <.001 | -.246 | .001 | -.329 | <.001 |
| Gender | -.029 | .64 | -.051 | .42 | -.068 | .30 | -.031 | .63 |
| Education | .128 | .07 | .135 | .05 | .075 | .30 | -.030 | .68 |
| Family Income | .073 | .26 | .078 | .24 | -.009 | .89 | .014 | .83 |
| Baseline performance | .057 | .47 | .053 | .52 | -.374 | <.001 | -.137 | .06 |
| Intellect LA | .138 | .03* | .125 | .06 | .157 | .02* | .118 | .08 |
|  | Reasoning Change  N=208 | | Speed Change  N=208 | | Memory Change  N=208 | | Vocabulary Change  N=208 | |
| Variables | R^2^= .23 f^2^=.29  F(6,201)= 10.122^a^ | | R^2^= .18 f^2^=.21  F(6,201)= 7.519^a^ | | R^2^=.11 f^2^=.12  F(6,201)= 4.317^a^ | | R^2^= .15 f^2^=.17  F(6,201)= 6.298^a^ | |
|  | ß | p-value | ß | p-value | ß | p-value | ß | p-value |
| Age | -.396 | <.001* | -.339 | <.001* | -.244 | .002* | -.344 | <.001* |
| Gender | -.035 | .57 | -.053 | .41 | -.073 | .27 | -.038 | .56 |
| Education | .127 | .08 | .138 | .04* | .100 | .17 | .025 | .73 |
| Family Income | .004 | .95 | .062 | .34 | -.018 | .78 | -.033 | .62 |
| Baseline performance | .106 | .19 | .078 | .35 | -.362 | <.001* | -.139 | .13 |
| Social LA | .148 | .02* | .145 | .02* | .075 | .27 | .130 | .05* |
|  | Reasoning Change  N=208 | | Speed Change  N=208 | | Memory Change  N=208 | | Vocabulary Change  N=208 | |
|  | R^2^= .21 f^2^=.26  F(6,201)= 9,109^a^ | | R^2^= .16 f^2^=.19  F(6,201)= 6.681^a^ | | R^2^=.11 f^2^=.12  F(6,201)= 4.157^b^ | | R^2^= .14 f^2^=.16  F(6,201)= 5.609^a^ | |
|  | ß | p-value | ß | p-value | ß | p-value | ß | p-value |
| Age | -.381 | <.001* | -.320 | <.001* | -.232 | .003* | -.324 | <.001* |
| Gender | -.039 | .54 | -.058 | .37 | -.076 | .25 | -.039 | .55 |
| Education | .147 | .04* | .159 | .01* | .108 | .13 | -.011 | .88 |
| Family Income | .051 | .43 | .060 | .36 | -.013 | .84 | -.003 | .95 |
| Baseline performance | .082 | .31 | .064 | .45 | -.369 | <.001* | -.119 | .115 |
| Physical LA | .054 | .39 | .054 | .41 | .037 | .58 | .018 | .79 |

*Legend:* LA: Leisure Activity; ß: standardized regression coefficient; ^a^*p* < 0.001; Gender: F = 0, M = 1; Family Income: <$75.000 = 0,

≥$75.000 = 1; didn’t know = 2 (10 participants). * p-values ≤ .05.

**Supplemental Table 5**

| Model 2 | Reasoning Change  N=140 | | Speed Change  N=140 | | Memory Change  N=140 | | Vocabulary Change  N=140 | |
| --- | --- | --- | --- | --- | --- | --- | --- | --- |
|  | R^2^= .24  F(7,120)= 6.692^a^ | | R^2^= .16  F(7,120)= 4.482^a^ | | R^2^=.13  F(7,120)= 2.580^a^ | | R^2^= .16  F(7,120)= 4.436^a^ | |
|  | ß | p-value | ß | p-value | ß | p-value | ß | p-value |
| Age | -.499 | <.001* | -.422 | <.001* | -.223 | .03* | -.372 | <.001* |
| Gender | -.031 | .69 | -.113 | .17 | -.090 | .30 | .078 | .35 |
| Education | .153 | .10 | .142 | .11 | .164 | .09 | -.009 | .92 |
| Family Income | .033 | .69 | .059 | .49 | -.071 | .43 | -.087 | .31 |
| Baseline performance | .003 | .97 | -.050 | .64 | -.401 | <.001* | -.196 | .03 |
| Thickness Change | .117 | .15 | .135 | .11 | -.104 | .24 | -.041 | .63 |
| Baseline LA | .182 | .02* | .190 | .03* | .051 | .57 | .100 | .24 |

*Legend:* LA: Leisure Activity; ß: standardized regression coefficient; ^a^ Significant model; Gender: F = 0, M = 1; Family Income:

<$75.000 = 0, ≥$75.000 = 1; didn’t know = 2 (10 participants). * p-values ≤ .05.

**Supplemental Table 6**

Absence of age moderation in the associations between leisure activity frequency and cognitive change

*Legend:* LA: Leisure Activity; ß: standardized regression coefficient; ^a^*p* < 0.001; Gender: F = 0, M = 1; Family Income: <$75.000 = 0, ≥$75.000 = 1; didn’t know = 2 (10 participants). LA.

| **Model 3A**  ***Age as continuous variable*** | **Reasoning Change**  **N=208** | | **Speed Change**  **N=208** | | **Memory Change**  **N=208** | | **Vocabulary Change**  **N=208** | |
| --- | --- | --- | --- | --- | --- | --- | --- | --- |
|  | R2= .25  F(7,200)= 9.560** | | R2= .20  F(7,200)= 7.302** | | R2= .12  F(7,200)= 4.116** | | R2= .16  F(7,200)= 5.608** | |
|  | ß | p-value | ß | p-value | ß | p-value | ß | p-value |
| Age1 | -.421 | <.001 | -.358 | <.001 | -.255 | .001 | -.348 | <.001 |
| Sex | -.026 | .68 | -.047 | .46 | -.065 | .33 | -.027 | .68 |
| Education | .130 | .06 | .141 | .03 | .088 | .22 | -.028 | .70 |
| Family Income | .060 | .34 | .067 | .30 | -.003 | .96 | -.008 | .90 |
| Baseline performance | .072 | .35 | .057 | .48 | -.363 | <.001 | -.129 | .08 |
| Baseline LA | -.028 | .90 | -.096 | .69 | .204 | .42 | .162 | .51 |
| Baseline LA * Age1 | .230 | .32 | .294 | .22 | -.074 | .77 | -.011 | .96 |
| **Model 3B**  ***Age as categorical variable*** | **Reasoning Change**  **N=208** | | **Speed Change**  **N=208** | | **Memory Change**  **N=208** | | **Vocabulary Change**  **N=208** | |
|  | R2= .22  F(7,200)= 8.298** | | R2= .18  F(7,200)= 6.542** | | R2= .12  F(7,200)= 3.918** | | R2= .14  F(7,200)= 4.863** | |
|  | ß | p-value | ß | p-value | ß | p-value | ß | p-value |
| Age2 | -.367 | <.001 | -.310 | <.001 | -.236 | .002 | -.317 | <.001 |
| Sex | -.027 | .66 | -.043 | .50 | -.064 | .34 | -.026 | .69 |
| Education | .091 | .20 | .120 | .07 | .071 | .32 | -.045 | .54 |
| Family Income | .046 | .47 | .057 | .38 | -.010 | .88 | .000 | .99 |
| Baseline performance | .119 | .12 | .099 | .22 | -.349 | <.001 | -.135 | .07 |
| Baseline LA | .112 | .57 | .044 | .83 | .221 | .30 | .225 | .28 |
| Baseline LA * Age2 | .080 | .68 | .147 | .46 | -.095 | .65 | -.083 | .69 |

**Supplemental Figure 1**

Confirmatory Factor Analysis diagram 1


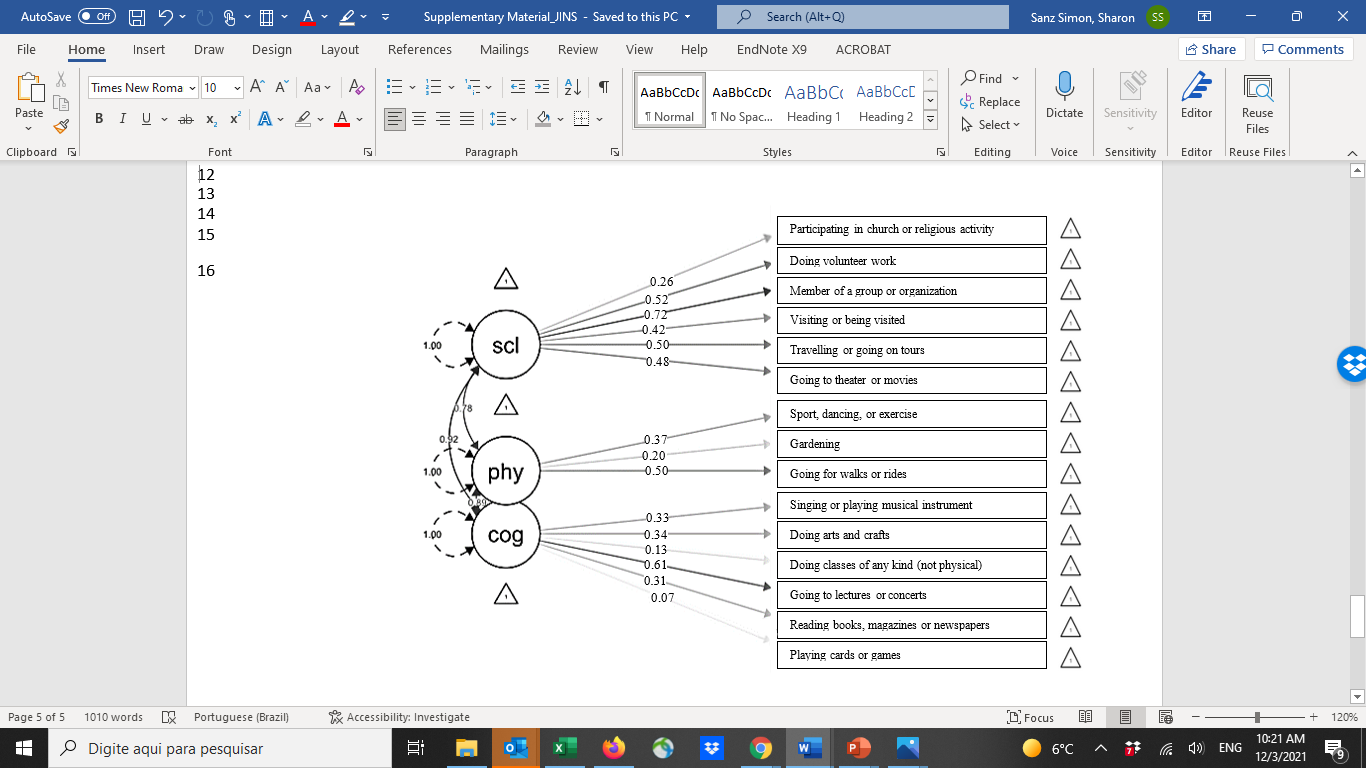


**Supplemental Figure 2**

Confirmatory Factor Analysis diagram 2

Model included only items with significant loadings

Note: Four LA items were excluded: “participating in church or religious activity”, “gardening”, “playing cards and games” and “doing classes of any kind (not physical)”


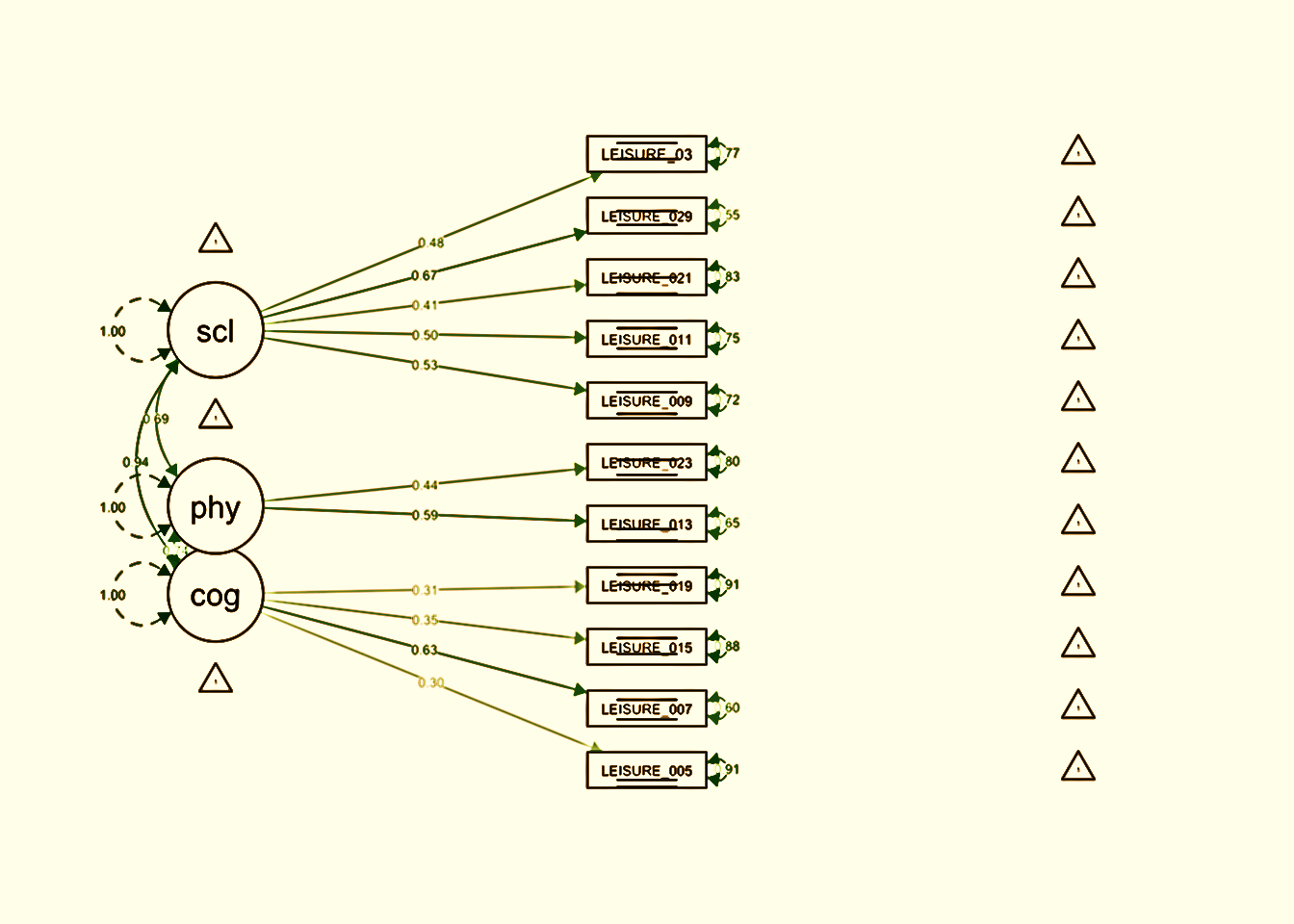

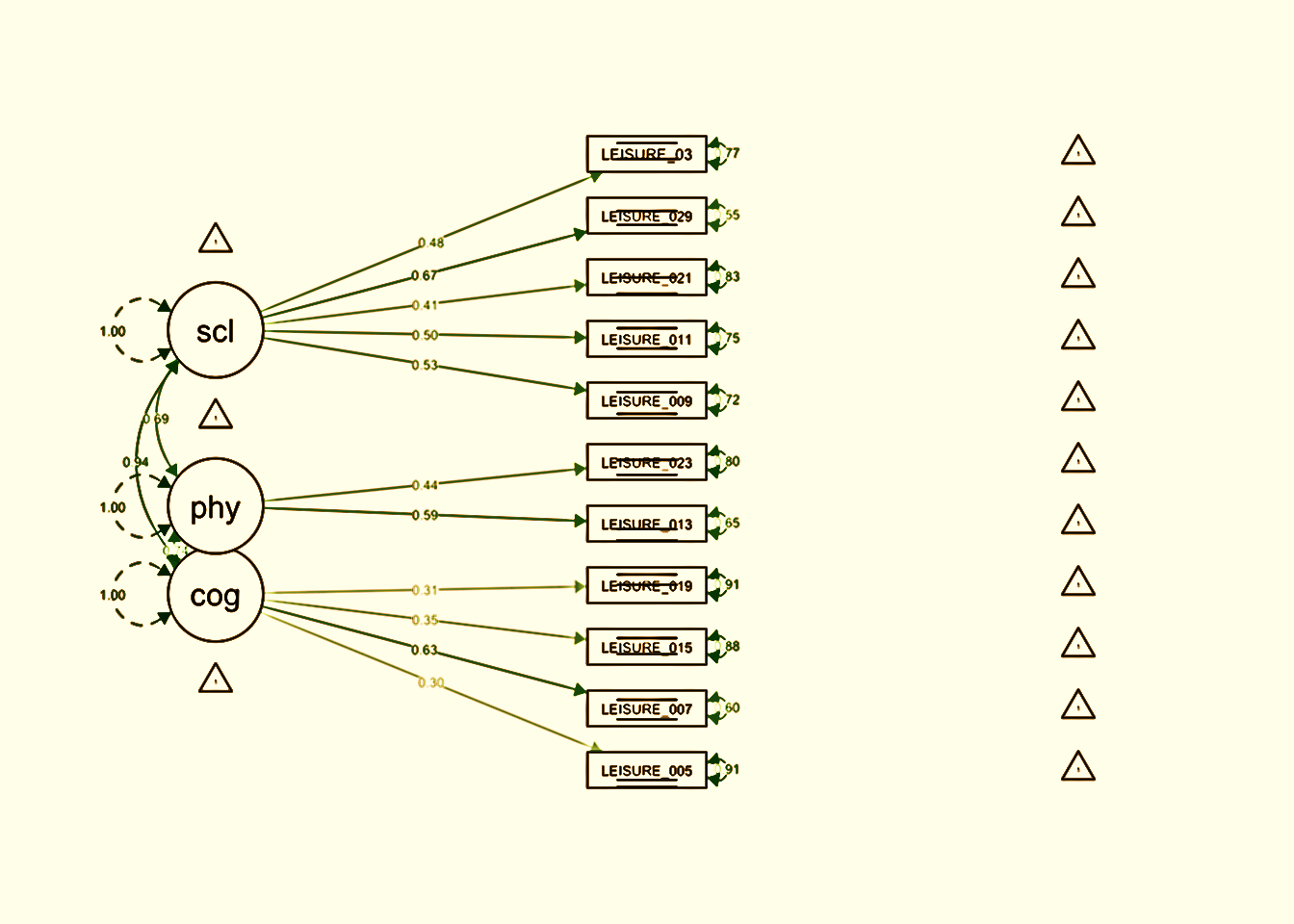


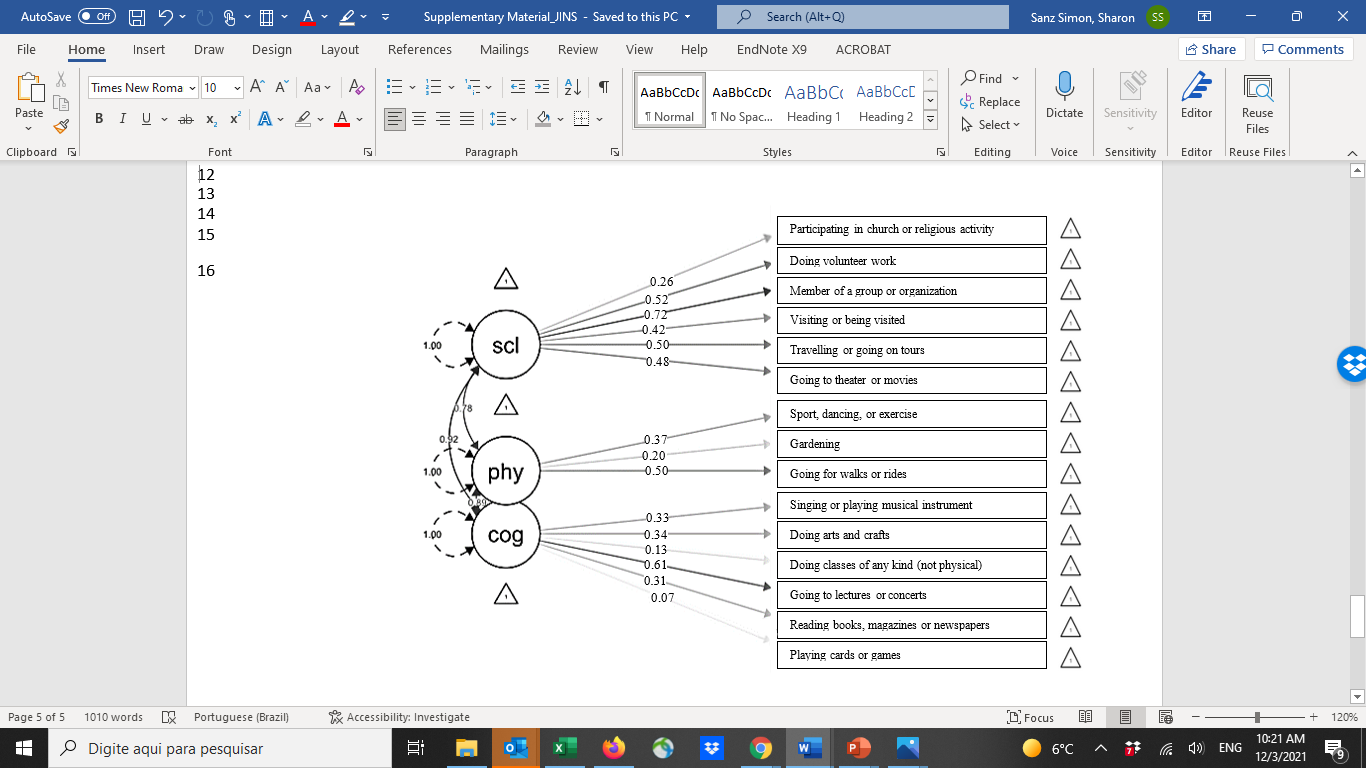


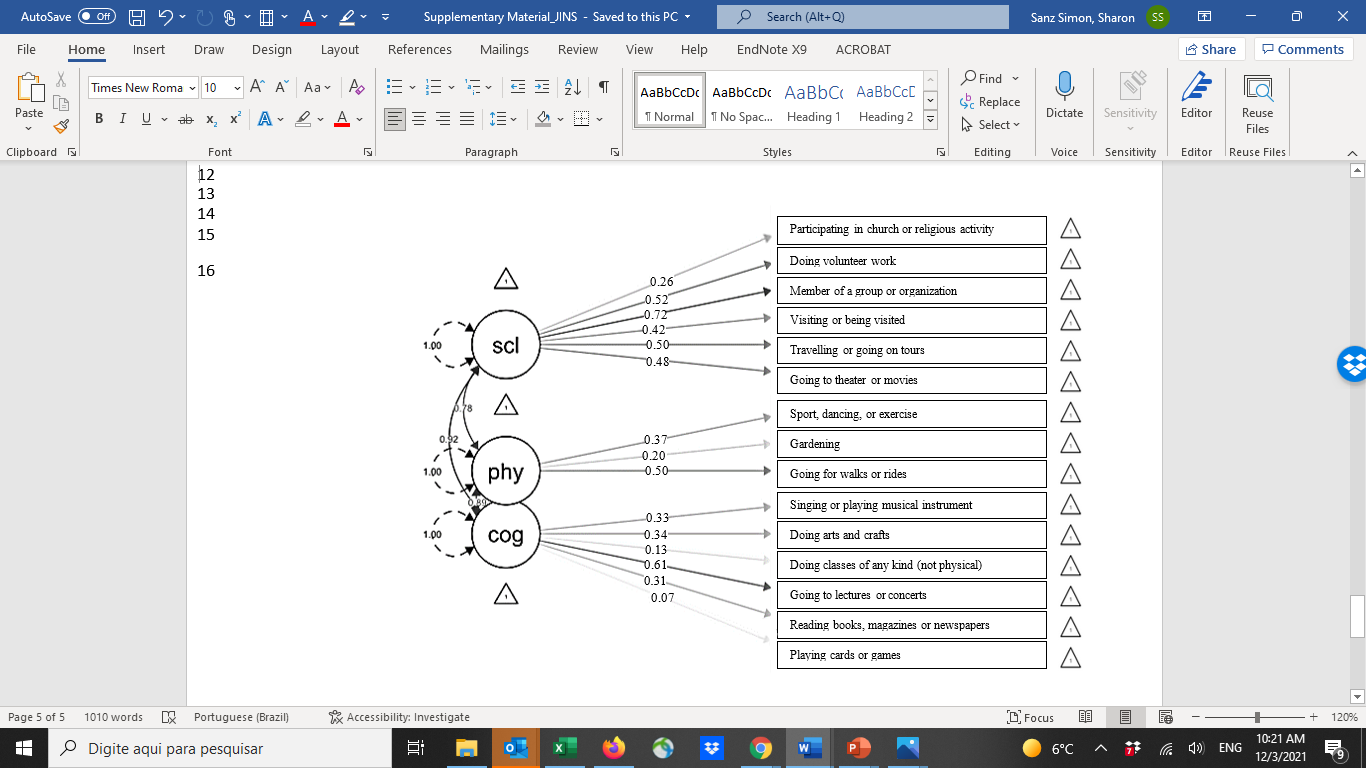

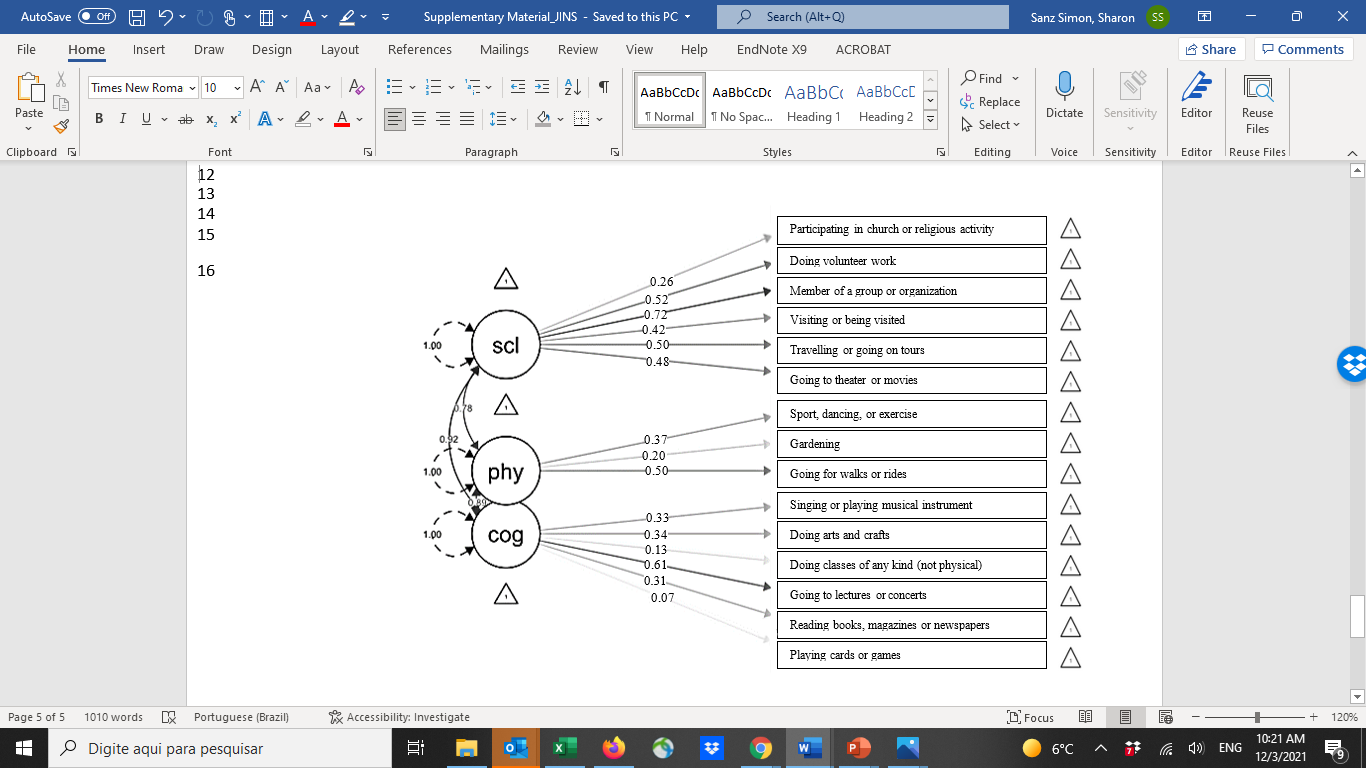

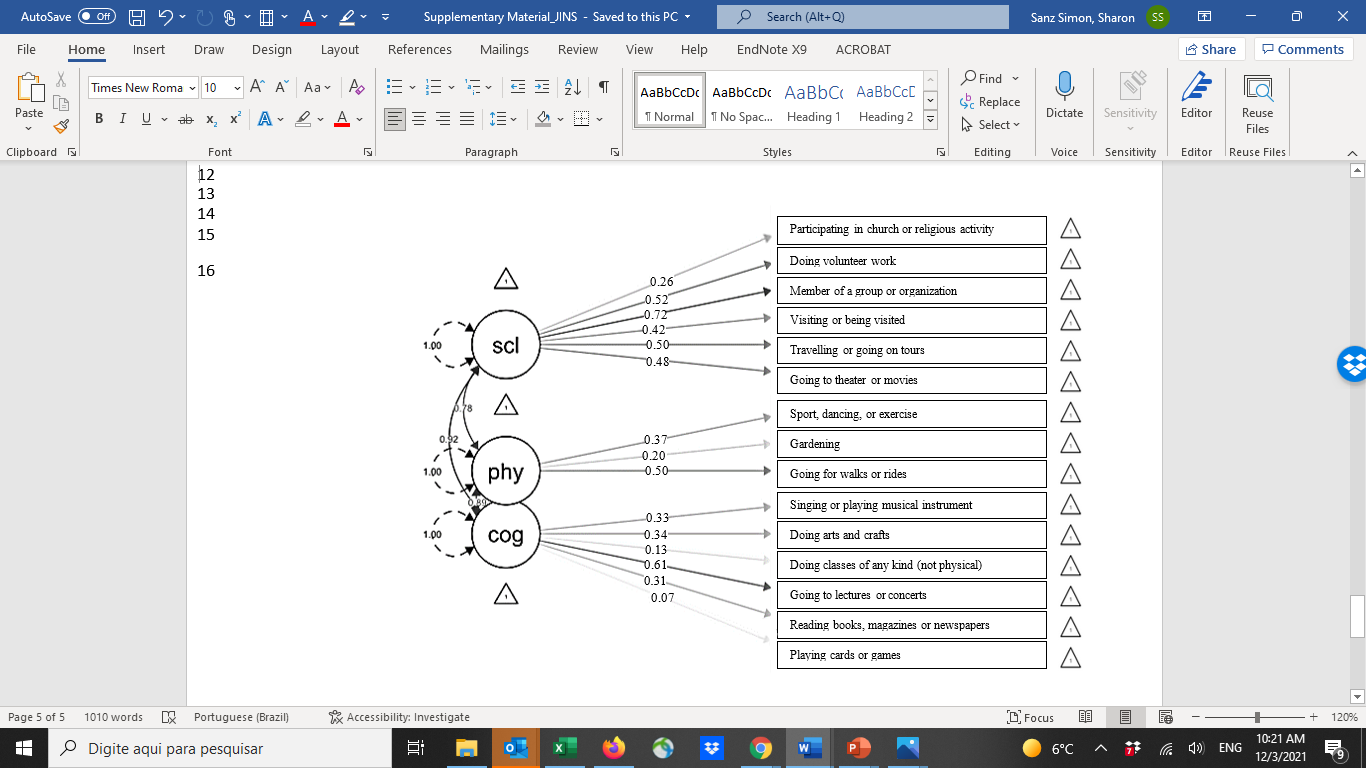

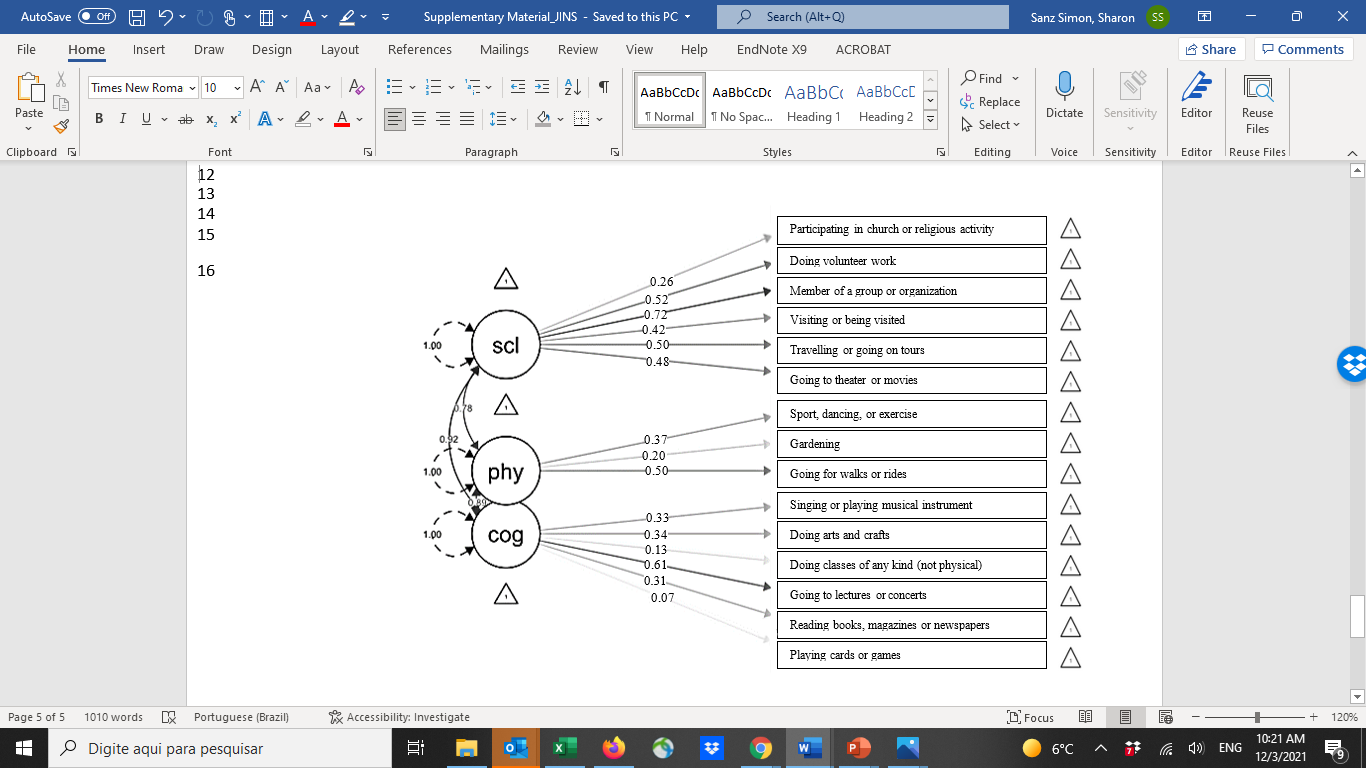

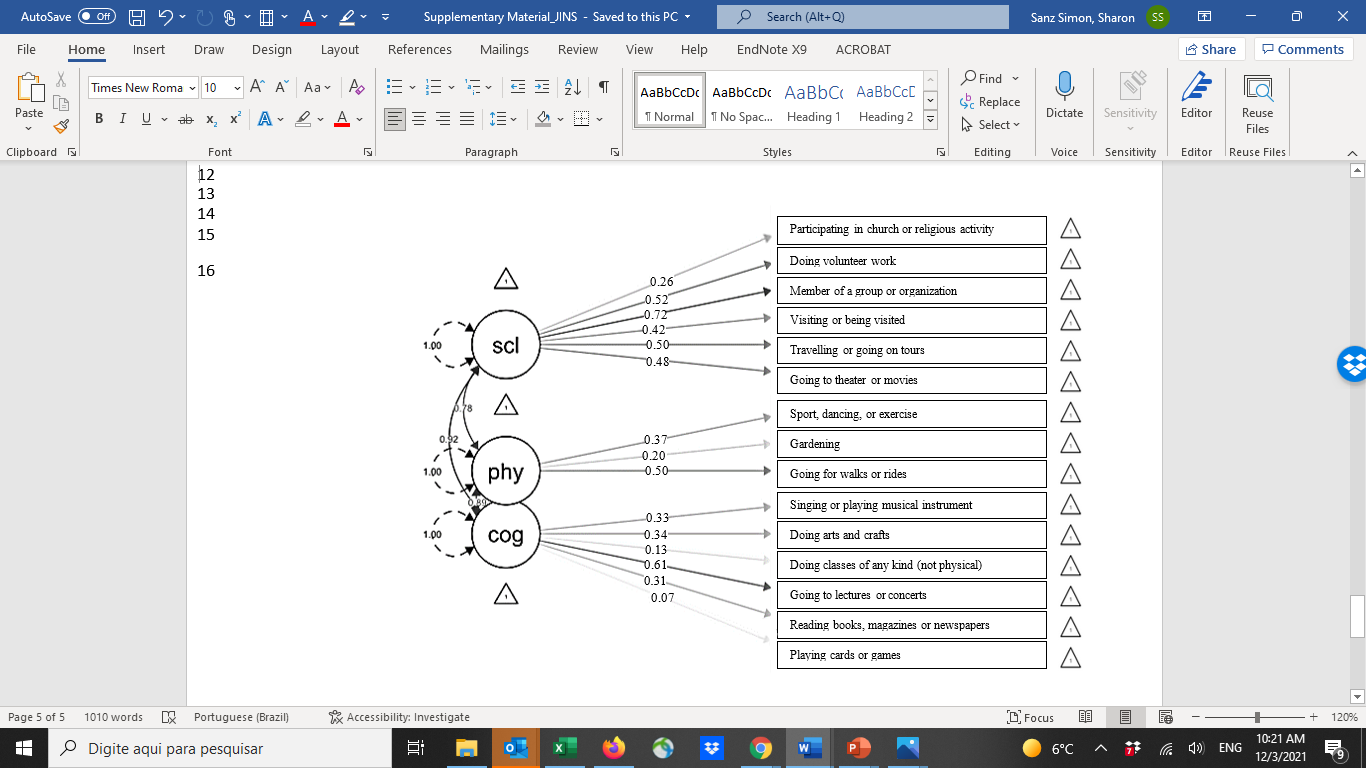

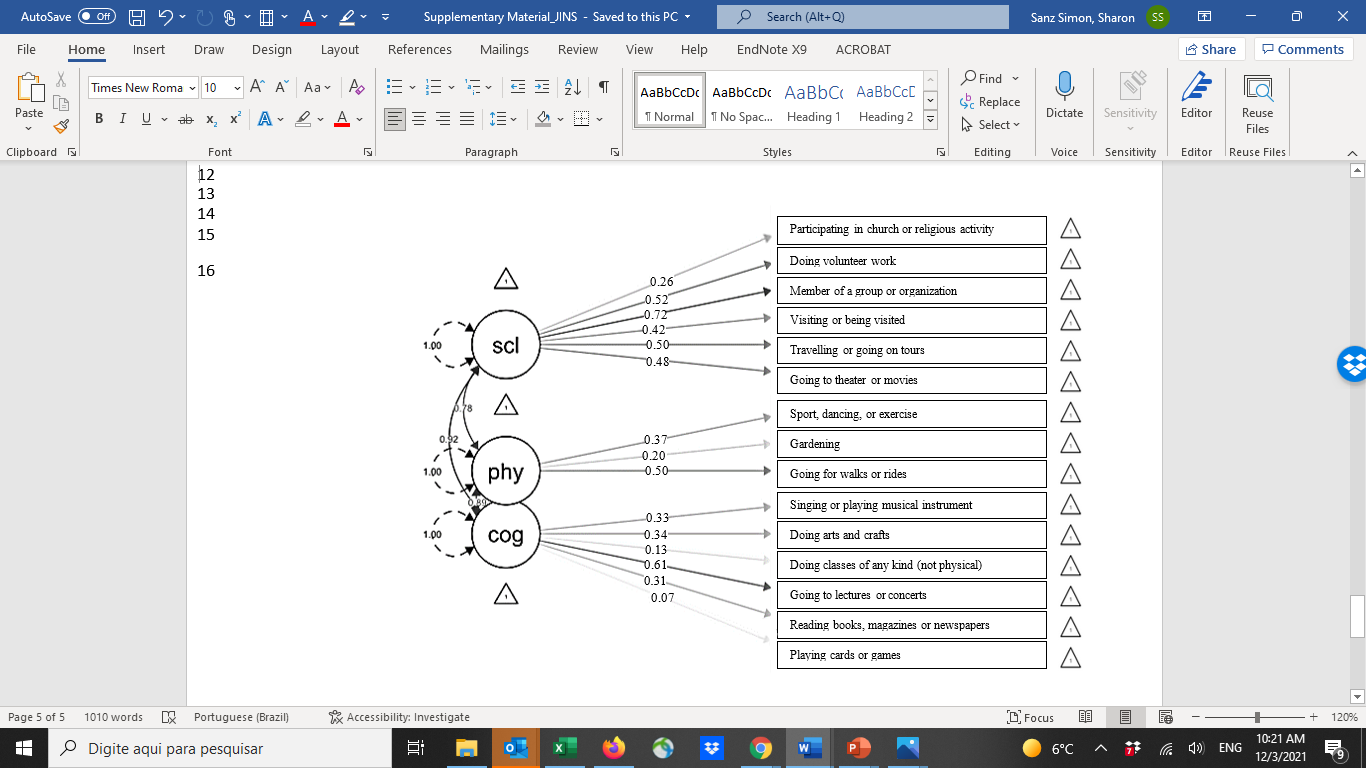

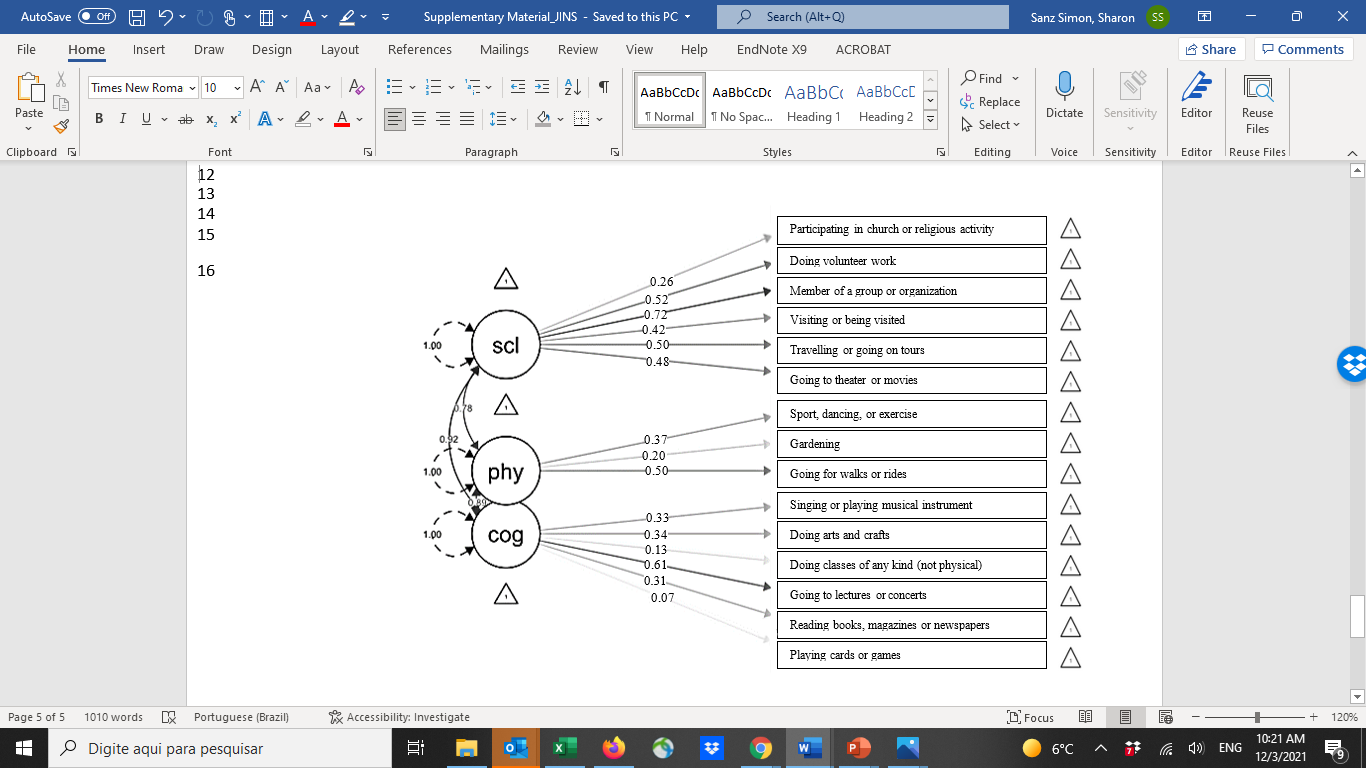

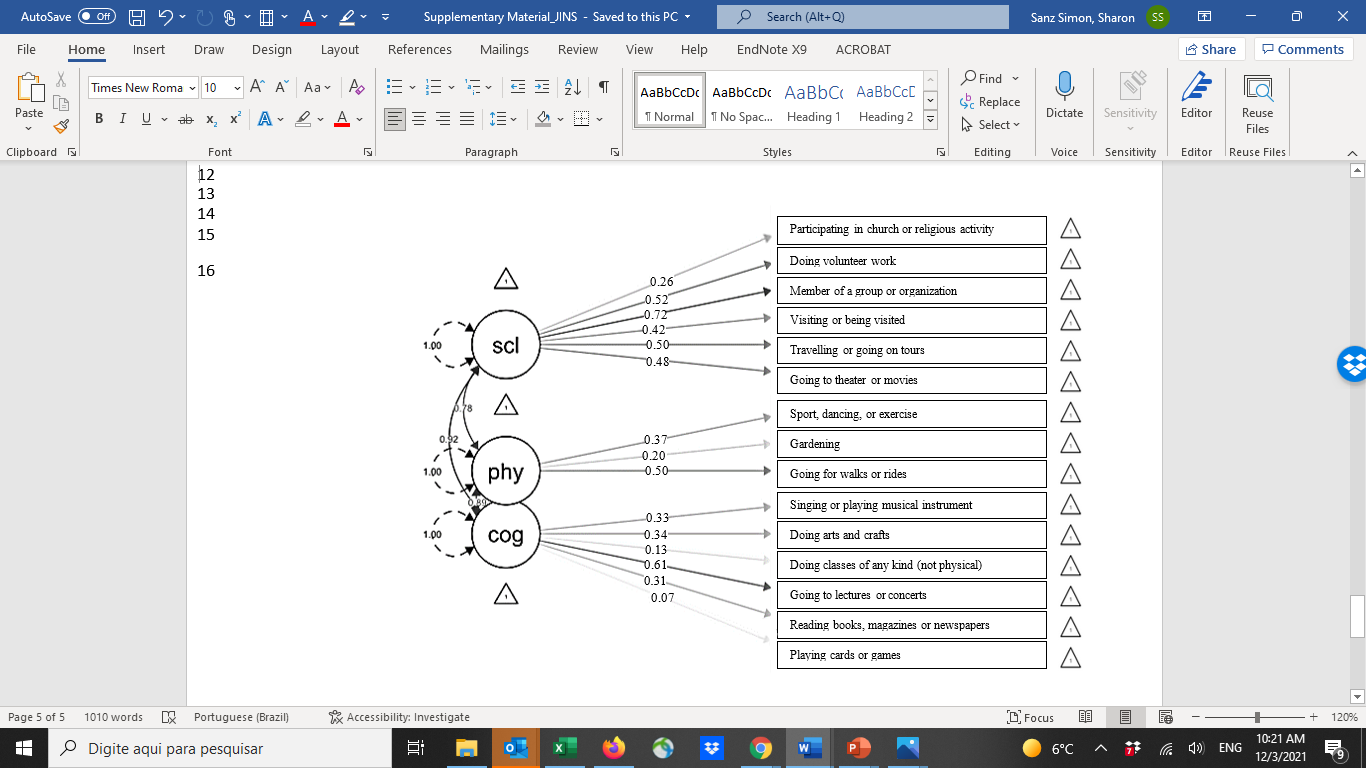

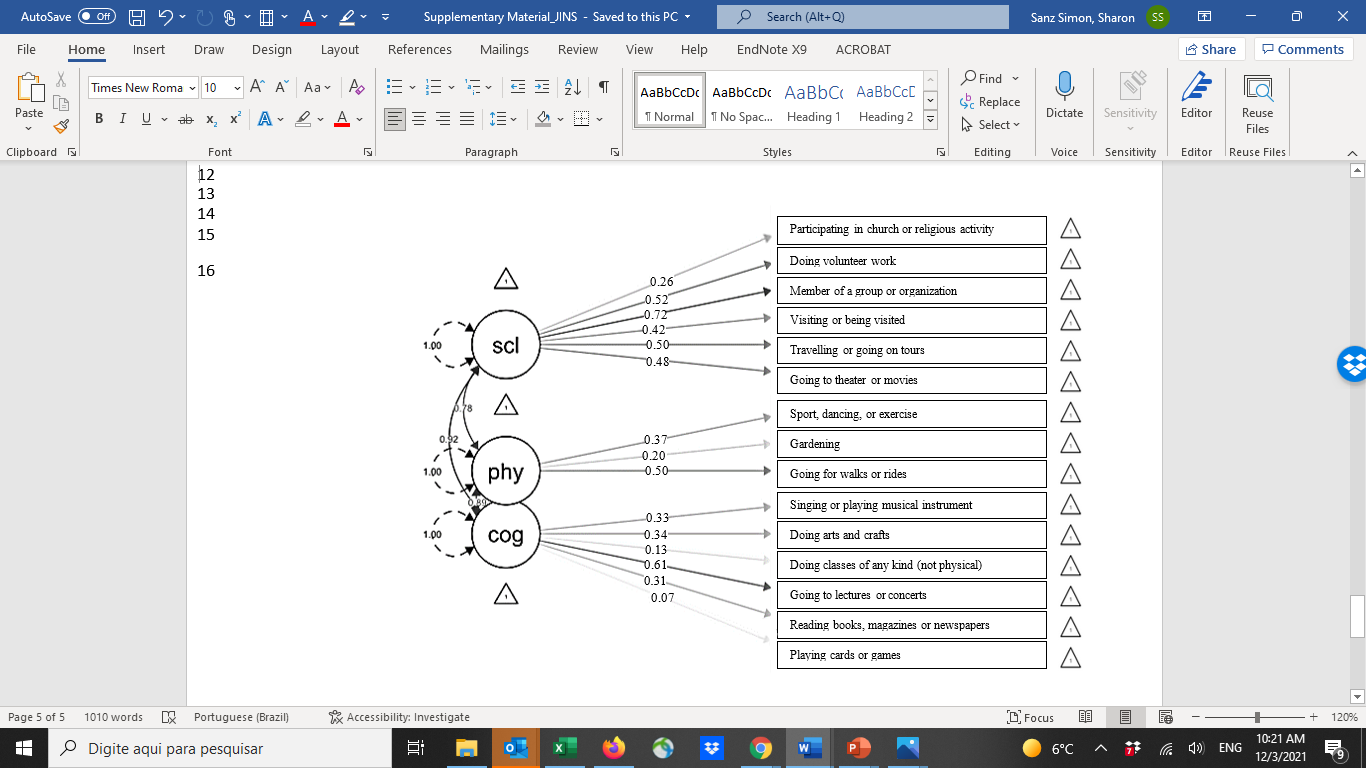

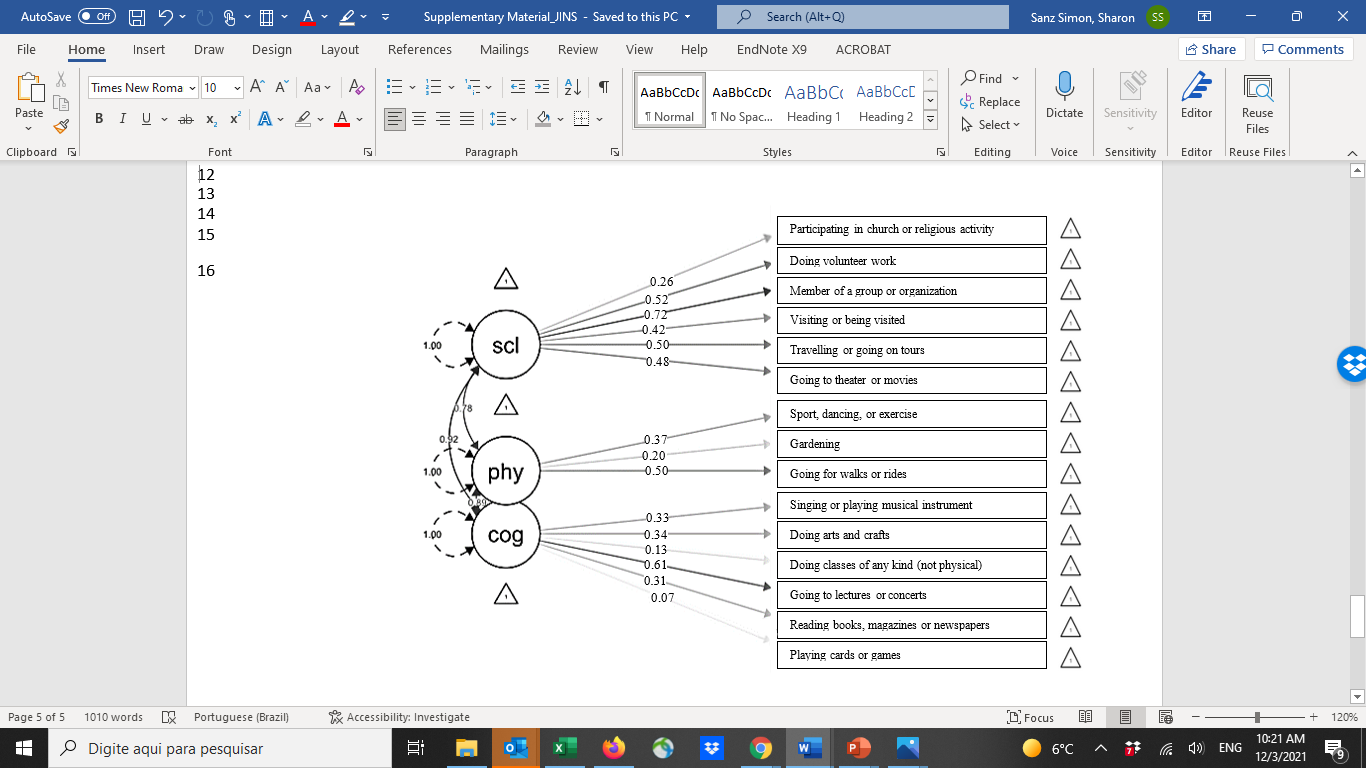

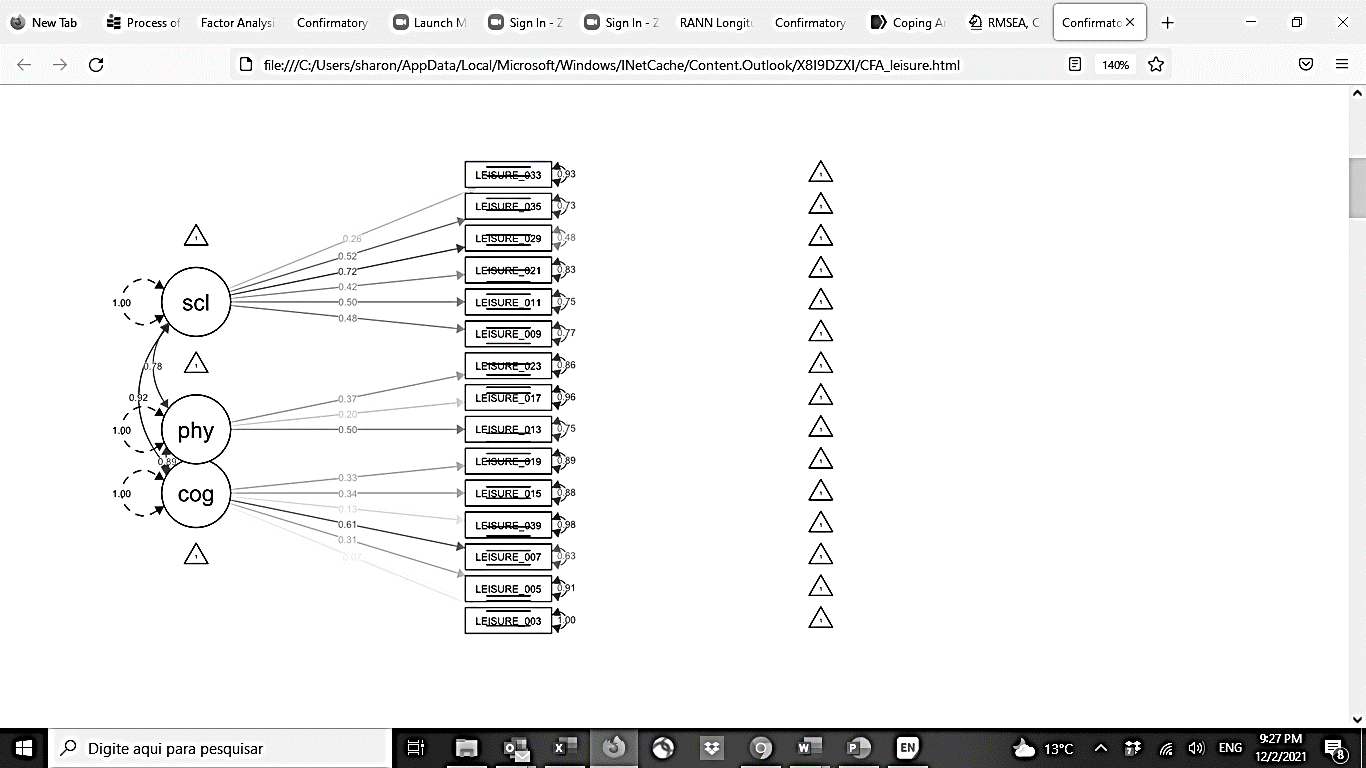

Supplement: Supplemental Material [file NIHMS1895065-supplement-Supplemental_Material.docx]
